# Supplementary material for: Accurate quantification of the stability of the perylene-tetracarboxylic dianhydride on Au(111) molecule–surface interface
Source: Commun Chem. 2023 Jul 3;6:136. doi: 10.1038/s42004-023-00925-2 (PMC10317958; doi:10.1038/s42004-023-00925-2)
Supplement: Supplementary file 2 — Supplementary information [file 42004_2023_925_MOESM2_ESM.pdf]

Supplementary Information for

Accurate quantification of the stability of the perylene-tetracarboxylic dianhydride on Au(111) molecule-surface interface

Victor G. Ruiz, Christian Wagner, Friedrich Maaß, Hadi H. Arefi, Stephan Stremlau, Petra Tegeder, F. Stefan Tautz, and Alexandre Tkatchenko*

*Corresponding authors.
E-mail: vicruiz85@gmail.com, alexandre.tkatchenko@uni.lu

This PDF file includes:

Supplementary text
Figs. S1 to S9
Tables S1 to S9
SI References

Supplementary Discussion

Temperature programmed desorption measurements

The relevance of thermal effects in the adsorption of molecules on surfaces must be addressed when a comparison between theory and experiment is put forward. It is clear that entropic effects can become quite important in the quantification of binding free energies of large molecules adsorbed on surfaces. The goal of this work is nevertheless, not to investigate thermal and entropic effects associated to the binding free energy of PTCDA on Au(111) or the comparison between theory and experiments at high temperatures or different coverages; but to establish a value for the adsorption energy that can be used as a benchmark quantity. It is for this reason that we do not focus on quantifying the free binding energy, but have chosen to quantify the adsorption energy at the limit of low temperature and zero coverage.

Our interpretation of the TPD experiments is based on transition state theory formulated via the Polanyi-Wigner equation Equation 3 and Equation 4 of the Supplementary Methods found in this supplementary information. Within this formulation, the energy term E_{Des} found in the exponential is not associated with a free energy but represents the activation energy of desorption which is equal to the adsorption energy under the assumption of a nonactivated process in which the adsorption/desorption process is reversible. Each TPD curve delivers one data point in an Arrhenius-like representation where the slope of this corresponds to the desorption energy E_{Des} as a function of coverage according to Equation 4. As a consequence, only a correction of up to $k_B T_{\mathrm{Des}}/2$ must be added to the final adsorption energy due to the gas impingement rate (1–3), which amounts to approximately 0.02 eV. This results in an adsorption energy of approximately 1.76 ± 0.10 eV extrapolated at low temperature according to the TPD experiments. From this perspective, the goal of our experimental setup is not to measure free adsorption energies but to focus on the adsorption energy of a single molecule, where the entropic effects are not large enough to substantially modify the comparison between theory and experiment.

Independently of these facts, it is nevertheless important to add that that entropic effects in large molecules become important when the molecule is highly flexible. In organic molecules, this happens mostly with the presence of single bonds that allow chemical functional groups or backbones in large molecules to rotate and wiggle due to thermal effects. An example of this can be found in the adsorption of a single azobenzene molecule on Ag(111) (4) or the adsorption of single oligophenyl molecules on amorphous silica (5). In contrast, PTCDA is not composed of single carbon bonds that can lead to flexibility, but a structure that is closer to nanographene, making it much stiffer than oligomers or olygophenyl molecules. Because of these reasons, we do not consider that entropic effects modify substantially the discussion of our results or the determination of the adsorption energy.

Low-temperature scanning probe microscope molecular manipulation experiments

In our previous work on determining the adsorption energy (and potential) from force gradient measurements, we have used a physically motivated model for the adsorption potential, implemented as a sum over parameterized atom-surface potentials summed over all atoms in the molecule. The parameterized potentials were functions of the atom-surface distance z and included exchange interactions (attraction and repulsion) as well as correlation interactions, i.e., vdW potentials (6). For the latter we used the asymptotic $(z - z_0)^{-3}$ expression valid for large z values. The parameters which define the molecule-surface potential have then been obtained by fitting simulated $\Delta f(z)$ curves for the lifting of PTCDA from Au(111) to the experimentally obtained curves.

With this modeling approach we employed the asymptotic $(z - z_0)^{-3}$ expression for the vdW potential even at $z < 4.5$ Å where it is not valid anymore (7) and where the actual vdW attraction increases slower than predicted by this asymptotic expression that diverges as z approaches the reference plane position z_0 (see purple curve in Figure S5). We avoided this problem in (6) by using $z_0 = 0$ instead of the actual value of $z_0 \approx 1.2$ Å such that the potential became shallower in the $z > 2.5$ Å region relevant to the adsorbed molecule. This effect is described in (7) where we have compared the adsorption potential in the vicinity of the surface to the long-range molecule-surface potential obtained with the correct z_0 value via a fit to experimental force gradient data of molecules fully detached from the surface.

In the present work, we performed a refined analysis of the experimental force gradient data of the lifting of

isolated PTCDA molecules from a reconstructed Au(111) surface at $T = 5\,\mathrm{K}$. We take the results of (6) and (7) into account and revisit the analysis of the short-range data with a more flexible model in order to fit the experimental data better and extract a more accurate corresponding value of the adsorption energy of PTCDA on Au(111) and the entire adsorption potential.

Molecular-mechanics modeling of the molecule-metal interface. When attempting to model the adsorption potential of an organic molecule on a noble metal, we are faced with the general problem that this potential will most likely be dominated by vdW interactions which, however, deviate for $z < 4.5\,\text{Å}$ in a non-trivial way from the known $(z - z_0)^{-3}$ behavior (7) (see Figure S5). While it is of course possible to obtain this potential from ab-initio calculations as discussed below, this would contradict the idea of a "measured" potential, i.e., a potential primarily obtained from experimental data. Hence, we choose an approach in which we abandon the limitations imposed by a *physically* motivated analytical potential (like $(z - z_0)^{-3}$) which simply does not exist for small z values, but instead model the vdW part of the adsorption potential as a generic function of z, specifically a cubic spline $E_{\mathrm{vdW}}(z) = Kz^3 + Lz^2 + Mz + N$. The short-ranged Pauli repulsion as well as a hypothetical chemical attraction between the carboxylic O atoms and the surface are, as in (6), approximated in a commonly used and physically sound way via parameterized exponential functions $E_{\mathrm{Pauli}}(z) = \exp(D - Az)$ and $E_{\mathrm{chem\ Ox}}(z) = -\exp(D - Az)$ where D and A are chosen independently for each atomic species and for the attractive and repulsive potentials respectively. Two such exemplary molecule-surface potentials are shown in Figure S5.

Since we know that the asymptotic expression for the vdW potential is valid at $z > 4.5\,\text{Å}$, we construct the spline such that it matches this asymptotic potential at $z = 4.5\,\text{Å}$ up to the second derivative (which corresponds to the force gradient, the quantity measured in our experiments). To make the spline flexible enough to emulate arbitrary potential profiles, we divided it into 9 segments of $\Delta z = 0.3\,\text{Å}$ width, such that a total of 9 parameters $K_1 \ldots K_9$ is required to describe the entire spline in the relevant range of $1.8\,\text{Å} < z < 4.5\,\text{Å}$. Note that, while each segment of the cubic spline is described by 4 parameters, the values of L, M, and N are defined automatically when requiring smoothness at the points were two segments meet. Different from the C_3 values of the asymptotic expression, the spline parameters have no eminent physical meaning. We account for the three different atomic species in PTCDA (C, H, O) by generating an individual spline for each species, using the asymptotic C_3 values known from experiment and simulation at $z = 4.5\,\text{Å}$ (7).

A second challenge in a molecular-mechanics simulation of molecular manipulation on a metal surface is a reasonably accurate modeling of the intramolecular mechanics. Here, we employ the model from (6) which has been designed with the idea of minimizing computational cost while reproducing the basic mechanical properties of the simulated molecule. To this end, costly non-bonding pair-potential terms are avoided such that the force-field is entirely based on bond, angle, and dihedral potential energy terms. The respective 33 force-field parameters were obtained by fitting to DFT (PBE+vdW)] (8) gas-phase energies for a training set of 1500 molecular conformations of PTCDA, each with an energy of less than $2.5\,\mathrm{eV}$ above the ground state energy (Figure S6). The conformations have been obtained by summing distortions corresponding to vibrational modes using random amplitudes. The overall stiffness is reduced in a second fitting step to account for the influence of the surface (see Supplementary Methods).

Another aspect that needs to be thought about when modeling molecular manipulation at the molecule-metal interface is the surface corrugation. The atomic structure of the surface leads to lateral variations of the molecule-surface potential. Since our modeling approach is based on atom-*surface* potential functions, the atomic structure of the surface is not automatically considered. While it could be added as an additional, laterally varying function (9), we have not pursued such an approach here as it adds more degrees of freedom (x, y, θ) to the simulation of the lifting process which, in our approach, has z as its single degree of freedom (see Supplementary Methods for the motivation behind this approach). An additional aspect which we have previously investigated (9) and now included into our model is an elastic tip apex, stretchable in z direction. During retraction of the tip and lifting of the molecule, forces of different magnitude act on the tip apex. The stiffness k_{tip} of the entire tip apex region and the corresponding elongation of the tip during lifting is also subject to fitting.

While the optimisation of the intramolecular force field (FF) is an important prerequisite, our actual goal is the recovery of the molecule-surface potential which fits best to our experimental $\Delta f(z)$ data. The fitting problem includes a total of 36 parameters, namely 11 parameters per atomic species, 2 additional values for the chemical bond

between the O_{carb} atoms and the Au surface, and finally the stiffness of the tip k_{tip}. Since an entire lifting process needs to be simulated to assess a single parameter set via its reduced χ^2 value, the search of this parameter space requires an elaborate approach.

For this, we have performed a Monte-Carlo sampling of possible adsorption potentials and the subsequent simulation of the lifting process for these potentials which yielded the best correspondence between simulation and experiment. The molecular mechanics lifting simulation is performed at $T = 0\,\mathrm{K}$, as described in (6, 7, 9). The tip is retracted in steps of $\Delta z = 0.025\,\text{Å}$ after each of which the molecule is relaxed into its local potential energy (PE) minimum conformation and the vertical force $F_z(z)$ on the tip is recorded. After completion of each lifting simulation $\Delta f(z)$ is obtained from $F_z(z)$ via a convolution step (10) that accounts for the fact that the experimental $\Delta f(z)$ data was obtained with the NC-AFM tip oscillating with an amplitude of approximately $0.5\,\text{Å}$. A detailed description of the generation of the parametrized potentials and of the simulation and fitting procedures can be found in the Supplementary Methods.

Another interesting result of our Δf fitting approach is the recovery of a distinct difference between the atom-surface potentials for carbon and oxygen atoms (Figure S1). This is enabled since we generate completely independent splines for the different atomic species and since the lifting process probes the atom-surface potentials of the two atomic species separately (oxygen atoms are the first and the last to be separated from the surface). The best fits plotted in Figure S1 feature O-Au(111) potentials which are deeper than their C-Au(111) counterparts and which also have a much lower equilibrium distance. With $\approx 0.55\,\text{Å}$, the latter difference is substantially larger than the differences in vdW radii of O and C atoms ($\approx 0.3\,\text{Å}$). Together, both effects indicate a considerable non-vdW contribution to the O_{carb}-Au(111) interaction. Our spline-based approach which is focused on the best-possible recovery of the *total* molecule-surface potential excludes the possibility to separate the individual contributions to the spline potential. For undercoordinated Au atoms as they are present at the SPM tip apex, covalent bonds between the carboxylic O atoms (O_{carb}) and Au are confirmed by the mere possibility to form a tip-molecule contact and lift the entire molecule from the surface using this bond. It is, however, important to note that the O-Au(111) interaction in Figure S1 is still quite weak (below $50\,\mathrm{meV}$ compared to hundreds of meV for the tip-O_{carb} bond (11)), such that other explanations for the observed effect become more likely. Such an explanation would be a rather strong electrostatic (charge-image charge) interaction due to the considerable negative partial charges on the O atoms of PTCDA (12). This interpretation is underpinned by the rather small determined difference in the atom-surface potentials of O_{an} (anhydride oxygen) and O_{carb} atoms, from which only the latter are in principle capable of forming chemical bonds. We model this difference by an attractive exponential potential applied only to O_{carb} atoms (see above) and for the best fits this potential is practically negligible in the relevant z-range (Figure S1). This absence of explicit O_{carb}-Au(111) chemical interaction is in accordance to our earlier findings (6).

Predictive first-principles modeling of complex inorganic-organic interfaces

Collective effects I: free-electron screening. The origin of dispersion interactions lies in the induced polarization resulting from collective instantaneous fluctuations of the electron density as each atom forming a molecule or a (complex) material responds to a dynamic internal electric field that depends on both local and non-local fluctuations associated with the surrounding atoms (13). The interface formed by organic/metal systems, being the result of the adsorption of organic aromatic molecules on a metal substrate, leads to the coupling of this (electronic) response of each system, a feature which is difficult to capture accurately in state-of-the-art electronic-structure models.

The description of dispersion interactions in the metal substrate has not been entirely successful so far due to the simultaneous presence of localized and delocalized (free) electronic states. Delocalized states or free-electron screening in metals give rise to a dynamically screened interaction between ions which effectively reduces the magnitude of dispersion interactions present in the adsorption of organic molecules on metal surfaces. This is a determinant factor in the resulting structure and stability of these interfaces.

The physical models underlying the first-principles simulation must be sufficiently accurate to capture the collective effects that lead to the emergence of screened dispersion interactions. It has been shown that this is crucial to predict the correct atomistic structure in organic/metal interfaces. The inclusion of free-electron screening is, in fact, crucial to predict the correct atomistic structure of complex inorganic/organic systems (14–16). Another consequence of

including the response of the substrate electrons in the determination of dispersion interactions in these systems that can be expected is an improvement in the quantitative prediction of the adsorption energy.

To investigate the adsorption geometry and energy of the PTCDA/Au(111) interface, we have modeled the system using the PTCDA surface density Θ which measures the number of adsorbed PTCDA molecules per surface unit cell. A full monolayer coverage is thus modeled by $\Theta = 1.0$ ML, which is defined as a herringbone lateral arrangement of two adsorbed PTCDA molecules per surface unit cell (see Supplementary Methods for details of the adsorption model). Figure S2(a) shows the adsorption system at $\Theta = 1.0$ ML. To calculate adsorption energies, we use two different definitions:

$$E_{\text{ads}}^{\Theta(\text{gas})} = -\frac{1}{N}\left[E_{\text{AdSys}}^{\Theta} - (E_{\text{Au}} + N \cdot E_{\text{PTCDA}}^{\text{gas}})\right], \quad [1]$$

$$E_{\text{ads}}^{\Theta(\text{ML})} = -\frac{1}{N}\left[E_{\text{AdSys}}^{\Theta} - \left(E_{\text{Au}} + E_{\text{PTCDA}}^{\Theta}\right)\right], \quad [2]$$

where $E_{\text{AdSys}}^{\Theta}$ is the energy of the adsorption system formed by PTCDA and the surface slab at a given molecule surface density Θ, E_{Au} is the energy of the surface slab, $E_{\text{PTCDA}}^{\Theta}$ corresponds to the energy of PTCDA at the given surface density Θ in periodic boundary conditions (PBC), $E_{\text{PTCDA}}^{\text{gas}}$ is the energy of a single PTCDA molecule in gas phase, and N corresponds to the number of adsorbed molecules in the system per surface unit cell. Equation 1 takes the isolated molecule in the gas phase as reference, so this definition contains contributions from molecule-metal *and* molecule-molecule interactions in the adsorption energy $E_{\text{ads}}^{\Theta(\text{gas})}$. On the other hand, Equation 2 takes a free-standing PTCDA surface density in PBC as reference, such that $E_{\text{ads}}^{\Theta(\text{ML})}$ contains only the contributions from adsorbate-substrate interactions as the formation energy of a molecular arrangement at surface density Θ, which is driven by attractive molecule-molecule interactions, is already included in the energetic term of the free-standing PTCDA surface density in PBC. Using the definitions given above, we have calculated the adsorption energy per molecule for two different molecule surface densities Θ of 1.0 and 0.5 ML after relaxation of the system. The resulting values for the adsorption energy using both definitions are shown in Table S1. The calculated energies reveal a dependence on the molecule surface density Θ that is independent of the reference state. In the case in which the reference is the free-standing PTCDA surface density, the adsorption energy $E_{\text{ads}}^{\Theta(\text{ML})}$ per molecule for $\Theta = 0.5$ ML *increases* with respect to the case of $\Theta = 1.0$ ML by around 6%. On the other hand, considering the formation of the PTCDA surface density from gas phase, $E_{\text{ads}}^{\Theta(\text{gas})}$ the adsorption energy per molecule for $\Theta = 0.5$ ML *decreases* with respect to $\Theta = 1.0$ ML by around 18%. These differences point to the fact that the PTCDA monolayer is formed via strong intermolecular forces as demonstrated in our TPD experiments in the monolayer regime (see inset of Figure 1A of the main manuscript). It is evident that these energies do not represent the single-molecule limit reproduced in either of our experimental results, which leads to the next step in the development of our first-principles model.

Coverage effects: extrapolation to the single-molecule limit. Taking the definitions given in Equation 1 and Equation 2 for the adsorption energy, the limit of low molecule surface density for both reference states should be virtually equivalent and the proper comparison to a single molecule result. Following our definition for $\Theta = 1.0$ ML, we have included calculations with Θ of 0.60, 0.45, 0.30, and 0.15 ML to obtain, via a systematic approach, the adsorption energy of the system in the single-molecule limit. For these results, we have modeled the system using a larger unit cell and a slab with fewer substrate layers (three layers) as described in the Supplementary Methods section and shown in Figure S2(b). The results are shown in Table S2 and Figure S3. They demonstrate that the adsorption energy tends to a converged value as the PTCDA surface density is reduced to the limit of the single molecule. Notably, at $\Theta = 0.15$, the difference between $E_{\text{ads}}^{\Theta(\text{ML})}$ and $E_{\text{ads}}^{\Theta(\text{gas})}$ amounts to just 0.04 eV. We take this value as the equivalent of the limit of low coverage in our calculations. Taking the average value between $E_{\text{ads}}^{\Theta(\text{ML})}$ and $E_{\text{ads}}^{\Theta(\text{gas})}$ at $\Theta = 0.15$ ML, the adsorption energy at the limit of the single molecule is 2.14 eV ($4 \times 4 \times 1$ k–point grid, see also the calculation details in the Supplementary Methods) with the PBE+vdW^{surf} method. Based on our calculations for $\Theta = 1.0$ ML reported on Table S1 which corresponds to a model with five metal layers for the substrate, this energy is underestimated by approximately 0.08 eV due to the number of substrate layers, resulting in an adsorption energy of 2.22 eV at $\Theta \approx 0.0$ ML.

Collective effects II: many-body dispersion effects. We have previously discussed (15, 17) how the variations of the electronic environment of each atom and the effect of dielectric screening in the dispersion interactions contained in the PBE+vdW^{surf} method cause a non-trivial reduction of the adsorption energy of the system. The DFT+vdW^{surf} method is, nevertheless, an effective pairwise approximation. Because of this fact, the adsorption energies predicted with this method tend to be overestimated due to the inherent absence of many-body effects in any effective pairwise model for the dispersion energy. In this regard, the full treatment of the collective response of the system, including many-body effects in the dispersion energy, represents an essential step to achieve quantitative accuracy in the prediction of adsorption energies of complex inorganic/organic systems. To overcome these limitations, our model includes many-body dispersion effects in the adsorption energy of PTCDA on Au(111) via the DFT+MBD method (18, 19). The MBD, as its formulation in terms of the random phase approximation shows, includes many-body effects in the long-range correlation energy to all orders and constitutes a very accurate technique when correct polarizabilities are used as input (19). We have calculated the adsorption energies of the system for the molecule surface densities of 0.5 and 1.0 ML, using the two different adsorption energy definitions discussed above, with the MBD method using the PBE XC functional (PBE+MBD). These results are also shown in Table S1. They demonstrate that including many-body effects in the dispersion energy reduces the magnitude of the adsorption energy by approximately 0.70 eV no matter the reference state or the coverage. MBD energies are calculated as a post-processing step in the PBE+MBD method, additional calculations at full monolayer coverage using the PBE+MBD-NL functional, also shown in Table S1, show a difference of approximately 10 and 30 meV for both definitions of the adsorption energy in comparison to PBE+MBD. These differences fall well inside any experimental errors. For comparison, the uncertainty interval of our TPD data is 200 meV and of the SPM-based data 500 meV.

To obtain the adsorption energy in the single-molecule limit having a consistent model, we have performed the same analysis for coverage effects that we did using the PBE+vdW^{surf} method. These results are summarized in Table S2 and shown in Figure S3. This analysis yields an adsorption energy of 1.59 eV per single molecule with the PBE+MBD method including the same correction due to the number of layers as mentioned above for the PBE+vdW^{surf} results. In comparison to the latter, this number shows that the inclusion of many-body effects in the dispersion energy reduces the magnitude of the adsorption energy by 0.63 eV.

Quantification of the self-interaction error in density-functional theory. The self-interaction of an electron with itself is one of the most fundamental deficiencies present in the approximations of the XC energy functional in DFT. It is known as self-interaction error (SIE) and is present in semi-local formulations for the XC functional such as PBE. It can lead, among other deficiencies, to a destabilization of localized orbitals, diminishing their binding energy (20). This effect can be of particular importance in the context of a first-principles prediction of the energetic stability of complex inorganic/organic interfaces.

To quantify the influence of the SIE in the adsorption energy of PTCDA on Au(111), we have performed single-point calculations of the adsorption energy at surface density $\Theta = 1.0$ ML, both with the DFT+vdW^{surf} and DFT+MBD methods, using a hybrid XC functional. Hybrid functionals combine a fraction of exact exchange defined in the framework of DFT with a fraction of semi-local XC to overcome the SIE. We have used the HSE XC functional (21, 22) given that it is based on a screened Coulomb potential for the exchange interaction, feature which makes it especially suitable for systems with metallic behavior. The resulting adsorption energies are shown in Table S3, where the value $\Delta_{\mathrm{HSE-PBE}}$ corresponds to the difference between the HSE and PBE calculations. By analyzing the differences between PBE and HSE, we can conclude that there is a systematic increase of approximately 0.25 eV due to the effect of the SIE, independent of the methodology to include dispersion interactions or the reference state for the adsorption energy.

Reconstruction of the* Au(111) *surface. The final step in our atomistic model is to consider the influence of the surface reconstruction on the adsorption energy of a PTCDA molecule. A structural model for the Au(111) consisting of a superposition of three 120° rotated domains was first proposed by interpreation of low energy electron diffraction (LEED) patterns (23). As an alternative, the surface structure can be described as a mixture of domains in which the majority corresponds to an fcc-top layer alignment and a smaller fraction which mostly aligns with hexagonal-close-packed surface sites (24). Experimental studies have further shown that this surface reconstruction is preserved upon

adsorption of a PTCDA monolayer (25–27).

We have performed a comparison of the contribution of dispersion interactions to the adsorption energy of a single adsorbed PTCDA molecule with the vdW^{surf} and MBD methods on; (a) the Au(111) surface and (b) the structure of the reconstructed surface taken from a previous work (24), which is optimized at the PBE level. Table S4 shows the mean and standard deviation of the dispersion energy $E_{\text{ads}}^{\text{dispersion}}$ (in eV) of 22 planar molecular configurations of a PTCDA molecule on both (a) and (b) substrates at a molecule surface density $\Theta \approx 0.09$ ML. In 11 of the molecular configurations, the molecular axis of the PTCDA molecule is aligned parallel to the $[110]$ direction of the substrate, shown in Figure S4(a) as Orientation 1. In the 11 complementing structures, the molecular axis of PTCDA is aligned parallel to the $[011]$ direction of the substrate (Orientation 2 in Figure S4(b)).

For each calculation, the molecule was placed at a height of 3.19 Å with respect to the average height of the reconstructed surface atomic top layer, while changing the position of the molecule in the direction parallel to the $[011]$ direction of the surface. The compression that exists in the top atomic layer of the reconstructured surface leads to a slight buckling of the top-layer gold atoms such that each surface gold atom is located at a different height. As a consequence, the height of the molecule changes depending on its particular position on the surface which results in a variance of the adsorption energy. Figure S4(c) and (d) show the variation of the adsorption energy with respect to the direction parallel to the surface area for Orientations 1 and 2, respectively.

In comparison, our structural optimization of the ideal Au(111) surface shows a negligible distortion of the surface top layer. Consequently, there are not variations in the adsorption energy that would depend on the particular adsorption position of the molecule on the ideal Au(111) (non-reconstructed) surface (see Table S4).

Following this procedure, we have found that the change in the *average* adsorption energy due to the reconstruction of the surface is not so large when compared to the ideal Au(111) surface, namely 0.06 and 0.04 eV without and with the inclusion of many body effects in the dispersion energy, respectively. The corrugation of the atomic top layer of the reconstructured surface introduces, however, a variation in the adsorption energy of ± 0.08 and ± 0.06 eV with and without the inclusion of many-body effects, respectively. This variance means that the adsorption energy falls within an interval of 0.16 and 0.12 eV with the vdW^{surf} and MBD methods, respectively.

Supplementary Methods

Temperature programmed desorption measurements

The desorption rate can be described by the Polanyi-Wigner equation (see Equation 3), with the coverage θ, the desorption order n, the pre-exponential factor (prefactor) ν, the desorption or binding energy E_{Des}, the temperature T, and the Boltzmann constant k_B:

$$\frac{d\theta}{dt} = -\nu(\theta) \cdot \theta^n \cdot exp\frac{E_{\text{Des}}(\theta)}{k_B T}. \quad [3]$$

A set of TPD curves with varying initial coverages (see Figure 1A of the main manuscript) allows to apply the so-called *complete analysis* introduced by King *et al.* (28), which is based on the Polanyi-Wigner equation (Equation 3). Apart from the Habenschaden-Küppers or *leading edge analysis* (29), for this method a guess for ν is not needed. Furthermore, it is the only method which allows analyzing measurements in order to determine the coverage dependency of E_{Des} (30). For a constant or given coverage θ_0, Equation 3 can be written in an Arrhenius-type form (see Equation 4, with n = 1), in which $ln\frac{d\theta}{dT}$ represents the normalized quadrupole mass spectrometer (QMS) intensity, and β the applied heating rate:

$$ln\frac{d\theta}{dT} = n \cdot ln\theta_0 + ln\frac{\nu(\theta_0)}{\beta} - \frac{E_{\text{Des}}(\theta_0)}{k_B T} \quad [4]$$

In each curve of a set of TPD spectra with different initial coverages θ_i, a given coverage θ_0 is reached at different temperatures, i.e., this temperature depends on the initial coverage of a measured TPD spectrum. Therefore each TPD curve delivers one data point in an $ln\frac{d\theta}{dT}[T(\theta = \theta_0, \theta_i)]$ *versus* $T^{-1}(\theta = \theta_0, \theta_i)$ Arrhenius-like representation (see inset of Figure 1B of the main manuscript). As Equation 4 shows, the slope yields the desorption energy as a function of coverage $E_{\text{Des}}(\theta_0)$. For $\theta_0 \to 0$ the desorption energy in the limit of single molecules can be elucidated

(see Figure 1B of the main manuscript). Note that the coverage dependence of desorption energies gives insights in lateral adsorbate interactions (31).

Low-temperature scanning probe microscope molecular manipulation experiments

As we have mentioned in the main text, the parameters which define the molecule-surface potential have been obtained by fitting simulated $\Delta f(z)$ curves for the lifting of PTCDA from Au(111) to the experimentally obtained curves. This method is favored over a simple double integration of the Δf, i.e., force gradient data for several reasons:

- The integration produces a potential energy profile along the trajectory of molecular conformations encountered in the manipulation, a collective coordinate of little meaning. We prefer $E(z)$ data for a molecule co-planar to the surface as usually computed.
- The surface corrugation has an effect on the force gradient data (9) which can be taken into account in the fitting procedure, but which would lead to artifacts in the potential curve obtained by double integration.

We now describe the model in detail as well as the fitting procedure to obtain the fit results shown in the main text.

Molecular-mechanics modeling of the molecule-metal interface. This reductionist approach can be motivated by several arguments:

1. We have only a very basic model of the surface corrugation at hand (9), which is of unknown accuracy and thus likely to induce errors.
2. At any given tip height the surface corrugation could cause an increase or decrease of the measured $\Delta f(z)$ beyond its value for an idealised "jellium" surface. Since our experimental data is an average over hundreds of lifting curves, each one potentially representing a different azimuthal orientation θ and position (x, y) of the molecule to be lifted, the respective deviations in Δf are likely to cancel to a large degree in the averaging process. Thus, the resultant averaged $\Delta f(z)$ curve is expected to be close to the ideal "jellium" case.
3. The z-regions in which the surface corrugation has a particularly strong effect on Δf can be clearly identified since there, the individual experimental curves $\Delta f(z)$ scatter strongly. Since we use the variance σ^2 of the experimental data as a weighting in the calculation of the reduced χ^2 used to measure the fit quality, the regions in which the corrugation is relevant is under-weighted in the fit, i.e., it will have a rather small impact on the outcome of the fit.

Generation of parameterized potentials, simulation, and fitting procedure.

DFT calculations. For all calculations (gas phase and adsorbed state) the PBE functional was used. For gas phase calculations, dispersion interactions were included via the Tkatchenko-Scheffler method (8) whereas the vdW^{surf} method (14) was used for the adsorbed state. The force threshold was 10^{-2} eV/Å and the SCF cycle convergence criteria for charge density, total energy and sum of eigenvalues were 1E-6, 1E-6 and 1E-3 respectively. The relaxations of the molecule in the adsorbed state were performed in two steps; pre-relaxation with light basis set (BS) followed by relaxation with tight BS. A 4-layer Au(111) slab (area = 23.125 $\times$ 20.027 Å^2) was used to simulate the surface and the lattice parameter were converged in advanced. A k-grid of $2 \times 2 \times 1$ was used to sample the BZ and minimum of 12 nm vacuum along z to avoid spurious interactions with periodic cell.

Intramolecular force field. The respective 33 force-field (FF) parameters were obtained by a Monte-Carlo search with search intervals slowly zeroing-in on a local optimum. In practice this has been achieved in a sequence of steps:

1. The FF parameters are initialised to some average plausible numbers (bonds: $1.4\,\text{Å}$, $k = 300\,\text{kcal/molÅ}^{-1}$; angles: $120\,°$, $k = 70\,\text{kcal/mol}$; dihedral angles: $180\,°$, $k = 4\,\text{kcal/mol}$). The C-H bond length is fixed to its actual value of $1.092\,\text{Å}$, since it has no interdependence with other parameters.

2. A sufficiently wide search interval around these initial values is defined (bonds: ± 0.1 Å, $\Delta k = \pm 150$; angles: $\pm 25\,^\circ$, $\Delta k = \pm 15\,\mathrm{kcal/mol}$; dihedral angles: $0\,^\circ$, $\Delta k = \pm 10\,\mathrm{kcal/mol}$, i.e., dihedral angles are fixed at a equilibrium value of $180\,^\circ$)

3. A Monte Carlo search is carried out, in which each parameter value is chosen randomly within the respective search interval. For each parameter set a χ^2 value is computed by summing squared errors $(E_{\mathrm{FF}} - E_{\mathrm{DFT}})^2/E_{\mathrm{DFT}}$ normalised to the DFT energy over all 1500 conformations in the training set. Normalisation is required to account for the large dynamic range of the energy values in the training set. Both, E_{FF} and E_{DFT} are calculated relative to the energy of the relaxed DFT gas-phase conformations ($E_{\mathrm{FF}} = \epsilon_{\mathrm{FF}} - \epsilon^0_{\mathrm{FF}}$ and $E_{\mathrm{DFT}} = \epsilon_{\mathrm{DFT}} - \epsilon^0_{\mathrm{DFT}}$). This implies the possibility that E_{FF} takes on negative values for some conformations if the FF happens to *not* have the DFT ground state as its lowest-energy conformation. To counter this effect, we penalize large FF energy values for the DFT ground state conformation by adding the respective energy value E^0_{FF} of the DFT ground state geometry to χ^2 using a weighting factor of 12:

$$\chi^2 = \sum_{i=1}^{1500} \frac{(E^i_{\mathrm{FF}} - E^i_{\mathrm{DFT}})^2}{E_{\mathrm{DFT}}} + 12\epsilon^0_{\mathrm{FF}}$$

This reduces "internal strain" in the FF and, since FF energies ϵ_{FF} can never be negative, will automatically reduce the possibility of negative E_{FF} values and generally the possibility that the molecule adopts false lowest-energy conformations during manipulation. In the final fit, only two out of 1500 E_{FF} values were negative ($-0.15\,\mathrm{kcal/mol}$ and $-0.3\,\mathrm{kcal/mol}$, respectively).

4. After each 150,000 Monte Carlo steps, the search intervals are adjusted. They are centered on the best values found so far and the widths of the search intervals are adapted to the magnitude of change in parameter value compared to the last search interval adjustment. In this manner, search intervals for parameters which remain almost unchanged (and thus are apparently converged) become narrower, while the other intervals remain wide. In this way, the dimensionality of the search problem is continuously reduced as more and more parameters converge and thus effectively drop out of the search.

The entire optimisation converged after 3.8 million Monte Carlo Steps. Several independent optimisation runs were made which all converged to similar χ^2 values, albeit with substantial variations in some FF parameters. This shows that the FF values are not independent and unique but that different parameter combinations can yield very similar E_{FF} values over the entire training set.

For the training set of 1500 PTCDA conformations, a cutoff for the DFT gas-phase energy of $E_{\mathrm{DFT}} < 2.5\,\mathrm{eV}$ is used. The accuracy of the FF model up to such rather high energies is needed even though the experiment is carried out at $T = 5\,\mathrm{K}$ where molecular vibrations are irrelevant, since strong forces are exerted on the molecule from tip and surface, bending it substantially. The purpose of the used FF model is not to accurately represent molecular vibrations but rather to reflect well the overall bending of the molecule in the junction between tip and surface, because this bending determines how the individual atoms of the molecule "sample" the molecule-surface potential. If, in a hypothetical example (32), the molecule was very stiff, all atoms would pass the steepest point of the adsorption potential almost simultaneously during lifting, which would cause a very strong molecule-surface force and make the molecule impossible to lift. An extremely floppy molecule, on the other hand, would be peeled off the surface like an adhesive tape, such that its atoms would pass the steepest point of the potential strictly sequentially (32).

A complication arises since the overall stiffness of the molecule is substantially reduced upon adsorption on the metal surface due to the increased delocalisation of its electrons. This effect can obviously not be captured in the *gas-phase* DFT calculations to which we fit the FF parameters. In a DFT calculation *including* the metal surface, on the other hand, this softening is likewise inaccessible because the actual adsorption energy is calculated by subtracting the energy of the isolated molecule (i.e. gas phase) such that any softening is summed into the adsorption energy. Our approximate solution to this problem is the introduction of a single factor f used to scale all 21 intramolecular FF parameters determining the stiffnesses of bonds, angles and dihedral angles. We obtained a value of $f = 0.5$ by comparing the PBE+vdW^{surf} *conformations* of a PTCDA molecule lifted from the Au(111) surface at three different

tip heights with the respective conformation in the FF simulation for a range of f values. Technically, this turns the entire optimisation into an iterative process, because a reasonable *molecule-surface* potential is required in the simulation of the lifting process to yield reasonable molecular conformations. We found, however, that a single iteration already yields a stable f value.

Molecule-surface potential. While the optimisation of the intramolecular FF is an important prerequisite, our actual goal is the recovery of the molecule-surface potential which fits best to our experimental $\Delta f(z)$ data. The fitting problem includes a total of 36 parameters, namely 11 parameters per atomic species ($K_1 \ldots K_9$, D, A), additionally 2 values for the chemical bond between the O_{carb} atoms and the Au surface, and finally the stiffness of the tip k_{tip}.

The vdW part of the molecule-surface potential are cubic splines:

$$E_{vdW} = (A_{X,n} \times z^3 + B_{X,n} \times z^2 + C_{X,n} \times z + D_{X,n}),$$

where $A_{X,n}$, $B_{X,n}$, $C_{X,n}$, and $D_{X,n}$ change their values in each of the $n = 10$ z-intervals of the spline and for each atomic species X = H, C, O.

The Pauli repulsion is modeled by an exponential

$$E_{Pauli} = \exp(E_X - F_X \times z),$$

with X = H, C, O, and the covalent interaction between O atoms and the surface is likewise modeled by an exponential

$$E_{Chem} = -\exp(G - H \times z).$$

The complete atom-surface potential function for H and C atoms is thus

$$E_{vdW} = (A_{X,n} \times z^3 + B_{X,n} \times z^2 + C_{X,n} \times z + D_{X,n}) + \exp(E_X - F_X \times z),$$

with X = H, C, and

$$E_{vdW} = (A_{O,n} \times z^3 + B_{O,n} \times z^2 + C_{O,n} \times z + D_{O,n}) + \exp(E_O - F_O \times z) - \exp(G - H \times z)$$

for O atoms. Since the spline segments are matched up to the second derivative at the points where they are connected, and since the first segment (at $z = 4.5$ Å) matches the asymptotic expression $E_{vdW} = C_3/(z - z_0)^3$, there are only nine free parameters per spline, leading to a total of 27 spline parameters. With 6 parameters for the Pauli repulsion and two for the O-Au attraction, this amounts to a total of 35 parameters of the molecule-surface interaction.

Since an entire lifting process needs to be simulated to assess a single parameter set via its reduced χ^2 value, the search of this parameter space requires an elaborate approach.

To obtain results with minimal bias, we again employ a Monte Carlo search, however, without dynamical narrowing of the search interval. The reason is that it makes no sense to attribute relevance only to a single best parameter set since we are fitting noisy experimental data with an approximate model (unlike the fitting for the intermolecular force field described above, where we used DFT ground-truth data assumed to be noiseless). Simply speaking, even a perfect fit does not necessarily have to be the *correct* result. Hence, we rather aim for identifying trends and investigate whether there exists a unique best *group* of potentials all having similar properties.

Since our model for the molecule-surface potential is extremely flexible and since testing a parameter set via the simulation of the manipulation process is computationally expensive, limitations to the sampled parameter space are required in order to sample physically plausible potential functions with a density that allows converging onto the best fits. Hence we employ a pre-screening to select only reasonable potential energy functions. We sample the parameter space in three batches, a broad search which samples the adsorption energy range homogeneously (100,000 samples), a search dedicated to map the low E_{ads} range (100,000 samples), and a third batch which is meant to identify the best parameter sets in a narrower, physically sensible search interval (400,000 samples). Since the "physically plausible" sampling in the first batch did not yield any potentials in the low-E_{ads} region, a separate sampling was performed there where the Pauli repulsion was allowed to exceed plausible values. This often lead to potentials with two minima and a bump around $z = 4$ Å (Figure 2C of the main manuscript), however, even somewhat unphysical potentials should be part of our unbiased search. The pre-screening conditions for the first two batches (termed "broad sampling" below) are identical (except for the Pauli repulsion), whereas the third batch ("narrow sampling") uses different conditions.

Pre-selection criteria for PE functions. The following bounds were used for the atom-surface PE function parameter space:

1. Spline generation: The spline parameters K_i are not selected randomly but rather follow a random walk with a

fixed maximal step size $-\Delta K < K_i - K_{i-1} < \Delta K$ with

$$\Delta K = \begin{Bmatrix} 0.26\,\mathrm{kcal/mol\AA}^{-3} & \text{broad sampling} \\ 0.06\,\mathrm{kcal/mol\AA}^{-3} & \text{narrow sampling} \end{Bmatrix}$$

2. For the exponential potentials $E_{\text{Pauli}}(z) = exp(D - Az)$ which approximate the atom-surface Pauli repulsion we choose A randomly with $A > 1$ and

$$A < \begin{Bmatrix} 21 & \text{broad sampling} \\ 11 & \text{narrow sampling} \end{Bmatrix}$$

The parameter D is selected randomly from an interval defined by the following two criteria

$$1\,\mathrm{kcal/mol} < E_{\text{Pauli}}(2\,\text{Å}) < 100\,\mathrm{kcal/mol}$$

$$E_{\text{Pauli}}(z = 4.5\,\text{Å}) < \begin{Bmatrix} 0.1\,\mathrm{kcal/mol} & \text{broad sampling} \\ 1.0\,\mathrm{kcal/mol} & \text{low-}E_{\text{ads}}\text{ sampling} \\ 0.1\,\mathrm{kcal/mol} & \text{narrow sampling} \end{Bmatrix}$$

For the narrow sampling the following criteria were imposed

$$E_{\text{Pauli}}(z = 2.5\,\text{Å}) > \begin{Bmatrix} 0.3\,\mathrm{kcal/mol} & \text{H atoms} \\ 0.7\,\mathrm{kcal/mol} & \text{O atoms} \\ 1.0\,\mathrm{kcal/mol} & \text{C atoms} \end{Bmatrix}$$

3. Specific checks are also imposed on the complete atom-surface potential energy $E_{\text{a}}(z)$ consisting of the vdW spline, the Pauli repulsion and the chemical bonding (for O_{an}) atoms and on the respective vertical force F_z:

$$E_a(3.3\,\text{Å}) < 0$$

$$\begin{Bmatrix} F_z(2.0\,\text{Å}) < 0 & \text{broad sampling} \\ E_{\text{a}}(2.0\,\text{Å}) > 1.5\,\mathrm{kcal/mol} & \text{narrow sampling} \end{Bmatrix}$$

4. Finally, the adsorption height has been restricted to a small region $z = 3.27 \pm 0.06$ Å around the experimentally known adsorption height of PTCDA on Au(111) (33).

5. The k_{tip} value was randomly selected from the interval $20 \pm 8\,\mathrm{kcal/mol\AA}^{-2}$.

Predictive first-principles modeling of complex inorganic-organic interfaces

Methods. Van der Waals interactions were included using $i)$ the DFT+vdW^{surf} method (14) and $ii)$ the many-body dispersion (MBD) method (18). $i)$ The DFT+vdW^{surf} method consists in the combination of the DFT+vdW method (8) for treating intermolecular interactions with the Lifshitz-Zaremba-Kohn (LZK) theory (34, 35) for the nonlocal many-body response of the substrate surface. This scheme adds the dispersion energy to the DFT total energy as a sum of $-C_6^{ab}R_{ab}^{-6}$ terms, where R_{ab} is the distance between atoms a and b. It includes, due to the LZK theory, the many-body response (screening) of the substrate electrons in the determination of the C_6 coefficients and vdW radii of the substrate atoms. The inclusion of these collective many-body effects, present within the substrate, in the determination of the vdW interaction goes beyond an atom-based pairwise description and yields a reliable description of bonding in organic-inorganic adsorption systems. $ii)$ The MBD method treats the long-range correlation energy by

making use of the random-phase approximation (RPA) in the dipole limit (36) based on a range-separation of the interelectronic Coulomb potential. The MBD, as its formulation in terms of the RPA shows, includes many-body effects in the long-range correlation energy to all orders and is a very accurate technique when correct polarizabilities are used as input (37).

In this work, we use the revised version (called MBD@rsSCS in (19)) which includes an improved description of highly anisotropic systems (19, 37). This method maps the atoms in the system onto a set of atom-centered quantum harmonic oscillators and use an effective oscillator Hamiltonian (18). This representation, however, assumes a finite electronic gap in order to divide the system into effective atomic fragments (18, 19), fact which does not provide an accurate description of delocalized electrons present for example in metallic systems. In order to circumvent this problem, we have used the vdW parameters of the DFT+vdW^{surf} scheme as input for the MBD method in order to approximate the effects of the collective behavior of delocalized electrons within the surface in the dispersion energy. We refer to this method as DFT+MBD, where DFT stands for the XC functional approximation that is being used.

An additional calculation of the adsorption energy for the system at full monolayer converage was performed using the MBD-NL XC functional (38). The MBD-NL functional is a general-purpose XC functional with the aim of unifying nonlocal vdW functionals for polarization and interactions methods for many-body interactions. The MBD-NL is a functional that can be applied to a wide range of molecular systems, including interfaces formed between organic molecules and inorganic materials.

Lattice constant. Two different lattice constants have been used for all calculations. The experimental lattice constant for Au was used to build the slab corresponding to the PBE+vdW^{surf} and PBE+MBD adsorption potential energy curves shown in Figure 2C of the manuscript. For all other calculations, including single point calculations with the MBD method and the HSE XC functional, the PBE lattice constant was used to build the surface slab. We have taken these values from previous work (1), corresponding to 4.062 and 4.159 Å for the experimental and PBE values respectively. According to the same work, the PBE+vdW^{surf} lattice constant is 4.163 Å, amounting to a difference of 0.004 Å between PBE and PBE+vdW^{surf}. The PBE+vdW^{surf} method simulates to some extent the screening effect by the metallic bulk electrons, but for the vdW energy inside the metallic bulk, this method rather overestimates the interactions between metallic electrons given that these are already described accurately by the PBE functional since PBE is reduced to the local density approximation (LDA) for homogeneous electron densities (1). It is because of this reason that we have chosen the PBE lattice constant to generate the surface slab in our calculations.

Adsorption potential energy curve. For these calculations, the interface consisted of a single molecule adsorbed on a Au(111) surface modeled with three metallic layers using the repeated-slab method. The surface unit cell was modeled with a $\begin{pmatrix} 6 & -6 \\ 1 & 10 \end{pmatrix}$ supercell and a vacuum width of 40 Å in order to minimize the interactions between neighboring molecules. The metal slab was generated using the experimental lattice constant of Au. Single-point energy calculations, without structural relaxation of the interface, were performed using a Monkhorst-Pack grid of $2 \times 2 \times 1$ k–points in the reciprocal space. Calculations using both the PBE+vdW^{surf} and PBE+MBD methods were done. A previous calculation (17) of the same curve using the PBE lattice constant used to generate the slab have shown a difference of 0.05 eV in the adsorption energy. We have shown in previous work (17) that PBE+vdW^{surf} adsorption energy values do not change considerably with respect to the lattice constant when the PBE lattice constant is used to generate the surface slab. Final adsorption energies were calculated using Equation 2 where $N = 1$.

Monolayer coverage model and additional calculation settings. PTCDA does not form commensurate monolayers on the Au(111) surface but rather exhibits a very close situation to a point–on–line growth on the $(22 \times \sqrt{3})$ reconstructed surface (25–27, 39), situation which is not accessible to any type of state of the art modeling. We have adopted the repeated-slab method generated with the PBE lattice constant of Au, which is of 4.159 Å in line with a previous investigation (1). To investigate the adsorption geometry and energy of a PTCDA monolayer, we have modeled the system using a $\begin{pmatrix} 6 & 1 \\ -3 & 5 \end{pmatrix}$ surface unit cell consisting of five layers each with 33 Au atoms as a reasonable approximation (see (40)). We have modeled the system using the PTCDA surface density Θ which measures the number of adsorbed PTCDA molecules per surface unit cell. A full monolayer coverage is thus modeled by $\Theta = 1.0$ ML, which is defined as a herringbone lateral arrangement of two adsorbed PTCDA molecules per surface unit cell.

Figure S2(a) shows the atomic structure of the interface at $\Theta = 1.0$ ML. We have performed a structural relaxation of the interface at $\Theta = 1.0$ ML using the PBE+vdW^{surf} method while fixing the Au atoms in the three bottom layers. The size of the vacuum gap was set to be approximately 50 Å. The structural relaxation was performed using a Monkhorst-Pack (41) grid of $4 \times 4 \times 1$ k–points in the reciprocal space. Final optimized structures were obtained employing *tight* settings in the FHI-AIMS code for all atoms, after an initial relaxation using *light* settings.

Final adsorption energies were calculated according to Equation 1 and Equation 2 by performing single-point calculations of the relaxed structures using a grid of $6 \times 6 \times 1$ k–points in the reciprocal space, *tight* settings for the basis sets of all atoms, and taking into account vdW interactions between metal atoms. Figure S7 shows the convergence of PBE+vdW^{surf} adsorption energies with respect to the k–point grid for $\Theta = 1.0$ ML, yielding converged energies within 0.5 meV using a k–point grid of $6 \times 6 \times 1$. MBD energies were calculated using a k-point grid of $8 \times 8 \times 1$ in the reciprocal space. Figure S8 shows the convergence of PBE+MBD adsorption energies with respect of the k–point grid for the MBD energies, using a $6 \times 6 \times 1$ k–point grid for the underlying PBE calculation. A numerical oscillation can be observed between k–point grids of $8 \times 8 \times 1$ and $10 \times 10 \times 1$, but a $8 \times 8 \times 1$ k–point grid yields adsorption energies within a precision of 0.1 meV.

To generate structures with lower molecule molecule densities and determine the influence of the number of layers in the relaxation, we have performed a relaxation of the interface at $\Theta = 1.0$ ML using the same surface unit cell but with surface slab consisting of three Au layers instead of five in which only the toplayer is allowed to relax. The resulting adsorption energies are shown in Table S5 and Table S6 for the PBE+vdW^{surf} and PBE+MBD methods, respectively. These results show a difference of 80 and 50 meV in the adsorption energy when the number of layers is increased from three to five with the PBE+vdW^{surf} and PBE+MBD methods, respectively. An additional single point calculation using a surface slab of six atom layers for the interface was also performed. These calculations take the five layers substrate interface structure as initial point, the resulting values are also shown in Table S5 and Table S6. The results show a difference between 10 and 30 meV when the surface slab is increased to six layers depending on the reference used, either the gas phase molecule or the free-standing monolayer, respectively, to calculate the adsorption energy. These results show that the number of substrate layers, k-point grid, basis set and their numerical settings, have been chosen to yield adsorption energies converged within 30 meV.

An additional calculation of the adsorption energy for the system at molecule surface density $\Theta = 1.0$ ML was performed with the PBE functional including dispersion interactions via the MBD-NL functional (38) as a comparison to the PBE+MBD adsorption energy where the MBD energies are calculated as a post-processing step. This calculation was performed using *tight* settings, a $6 \times 6 \times 1$, and a $8 \times 8 \times 1$ k-point grids for the PBE and MBD-NL corresponding parts of the calculation, respectively. The structure for this single-point calculation corresponds to the PBE+vdW^{surf} relaxed system at $\Theta = 1.0$ ML using *tight* settings and a model substrate of five metal atom layers, where the three bottom layers were fixed during relaxation.

Calculations for the system at a molecule surface density $\Theta = 0.5$ ML were performed using the same procedure and computational settings, using an arrangement of one PTCDA molecule per surface unit cell taken from the herringbone lateral arrangement of the system at full monolayer coverage. Figure S2(a) shows the atomic structure of the system.

Model systems for lower molecule surface density and additional calculation settings. We have calculated the adsorption energy of the system at different molecule surface densities, namely with Θ of 0.60, 0.45, 0.30, and 0.15 ML. For the calculations, we have modeled the system with a larger surface unit cell with area of 824 Å^2 and a slab consisting of three layers each with 110 atoms of Au. The surface unit cell in this model is rectangular with unit cell vectors of magnitude a = 32.352 Å and b = 25.471 Å. At $\Theta = 0.60$, the coverage corresponds to the adsorption of four molecules in a herringbone arrangement on top of the surface unit cell, thus being consistent with our above-given definition of monolayer coverage ($\Theta = 1.0$). PTCDA surface densities of 0.45, 0.30, and 0.15 ML thus correspond to the adsorption of 3, 2, and 1 molecule(s) on the same surface unit cell. For these calculations, we chose the size of the vacuum gap to be approximately 50 Å and a Monkhorst-Pack (41) grid of $2 \times 2 \times 1$ k-points in the reciprocal space. We performed the structural relaxation of the system using the PBE+vdW^{surf} method only at $\Theta = 0.60$ ML by fixing the Au atoms in the two bottom layers. For this structural relaxation, we did not take into account vdW interactions between metal atoms and used *light* settings. The remaining model systems with molecule

surface densities Θ of 0.45, 0.30, and 0.15 ML were built using the system at $\Theta = 0.60$ ML as a starting point. Van der Waals interactions were taken into consideration only in the calculation of the final adsorption energies using *tight* settings and Eqs. Equation 2 and Equation 1. A single-point calculation of the adsorption energy at $\Theta = 0.15$ ML with $4 \times 4 \times 1$ k–point grid for the PBE part of the calculation shows that adsorption energies are converged within an accuracy of 10 meV as we show in Table S7. Final corrected energies at the limit of a single molecule ($\lim \Theta \to 0$, Table S2) correspond to the average of the adsorption energies calculated with Equation 1 and Equation 2 at $\Theta = 0.15$ ML using a k–point grid of $4 \times 4 \times 1$ and a correction due to the number of substrate layers as explained in the main text.

Self-interaction error quantification. To quantify the influence of the self-interaction error in the adsorption energy of PTCDA on Au(111), we have performed single-point calculations of the adsorption energy at surface density $\Theta = 1.0$ ML using the HSE hybrid XC functional (21, 22), at both levels of theory to include vdW interactions: HSE+vdW^{surf} and HSE+MBD. Due to the computational cost of a hybrid functional such as HSE, these calculations were performed using *light* settings, a $4 \times 4 \times 1$ k–point grid , and a model substrate with three metal atom layers built with the PBE lattice constant of Au. The resulting adsorption energies were compared to PBE+vdW^{surf} and PBE+MBD calculations using the same computational settings. The structure for this comparison corresponds to a PBE+vdW^{surf} relaxation of the system at $\Theta = 1.0$ ML using *light* settings and a model substrate of three metal atom layers generated with the PBE lattice constant, where the two bottom layers were fixed during relaxation.

Influence of the reconstructed surface on the single molecule adsorption energy. We have calculated the adsorption energy of a single PTCDA molecule adsorbed taking into account only the contribution of dispersion interactions with the vdW^{surf} and MBD methods. The atomistic model of the $(22 \times \sqrt{3})$ reconstructed Au(111) surface slab, which is also optimized at the PBE level, was taken from the work found in Ref. (24). 22 planar molecular configurations of a PTCDA molecule on Au(111) at a coverage $\Theta \approx 0.09$ ML were calculated as shown in Figure S4 and Table S4. These energies were calculated via

$$E_{\text{ads}}^{\text{dispersion}} = -\left[E_{\text{AdSys}}^{\text{dispersion}} - \left(E_{\text{Au}}^{\text{dispersion}} + E_{\text{PTCDA}}^{\text{dispersion}}\right)\right] \qquad [5]$$

where $E_{\text{AdSys}}^{\text{dispersion}}$ is the dispersion energy of the complete system formed by the adsorbate and the slab, $E_{\text{Au}}^{\text{dispersion}}$ is the dispersion energy of the slab, $E_{\text{PTCDA}}^{\text{dispersion}}$ corresponds to the dispersion energy of the adsorbate in periodic boundary conditions. Due to the low coverage, these systems correspond approximately to the limit of a single adsorbed molecule.

The MBD energy calculations for these cases were performed using PBE Hirshfeld weights as input and a k–point grid of $8 \times 8 \times 1$ for the calculations. Figure S9 shows the numerical convergence of dispersion adsorption energies with respect to the k–point grid, which shows that an $8 \times 8 \times 1$ k–point grid yields converged energies within approximately 1 meV.

Influence of stress on the surface area of the Au(111) surface on the adsorption energy. Experiments performed using x-ray diffraction (42, 43) to study the thermal behavior of the Au(111) surface between 300 and 1250 K show that there is a phase transition occurring between 865 and 880 K which yields a partially disordered fluid phase that exists beyond 880 K (42). At $T < 865$ K, the surface structure consists of an equilibrium density of kinks between rotationally equivalent domains of the $(22 \times \sqrt{3})$ reconstruction. The $(22 \times \sqrt{3})$ structure itself consists of discommensurations that separate regions with fcc stacking from regions with hcp stacking (42). Therefore, the domains of the reconstructed surface can be described by a kink periodicity and a discommensuration periodicity. As the temperature increases, the experiments have shown that both periodicities evolve to smaller values (42, 43) and the area per atom decreases slightly. However, they also found that the separation between the top two layers is expanded 3% relative to the bulk (111) interlayer spacing. Equally important is to mention that, the ratio between fcc and hcp stacking is maintained between 300 and 1250 K according to experiments (42) and that the adsorption domains of PTCDA are far larger than the surface reconstruction domains (27).

With these experiments in mind, two different adsorption system geometries were prepared to investigate the effect of a possible expansion (compression) of the surface unit cell. Employing our results for the adsorption system at molecule surface density $\Theta = 0.5$ ML, we have modeled two systems where the surface unit cell is either expanded or compressed by approximately 5% with respect to the ideal Au(111). We have performed a structural optimization with the PBE+vdW^{surf} method of both systems. Table S8 shows the results, including the distance of the molecule with respect to surface toplayer, the input expansion or compression (ΔA_{111}) of the surface unit cell with respect to the ideal (111) surface, and the resulting expansion or compression of the top two layers with respect to the ideal (111) surface (Δd_{12}).

The results show that the system is more sensitive to a compression in the surface area which results in an expansion of the distance between the two top layers that is larger relative to the case in which the surface area is subject to an expansion (5.2% against -2.8% in Table S8). To investigate the effect of this in the adsorption energy of the monolayer, we show the theoretical adsorption energies in Table S9. These results show that the effect of a compression or expansion in the surface unit cell has, in general, a small effect on the adsorption energy. In particular, many-body effects in the dispersion energy are not affected by the expansion or compression of the surface area in the adsorption system.

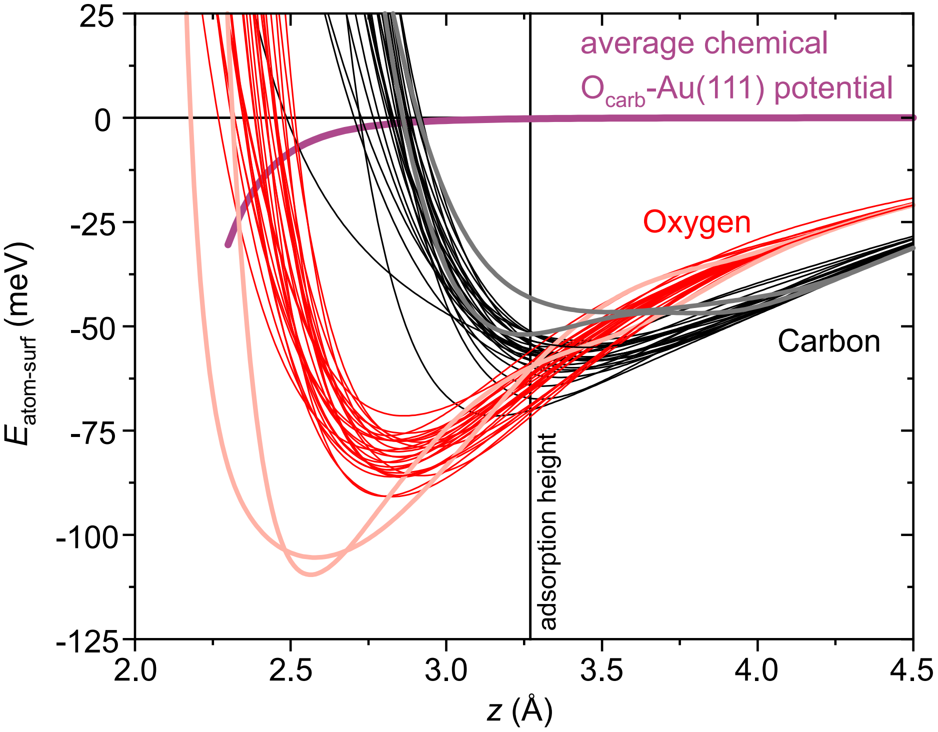

Fig. S1. Atoms-surface potential fit results. Plot of the atom-surface potentials of carbon and oxygen for the best fits ($\chi^2 < 2.3$, see Figure 2 of the main manuscript). Both sets of curves exhibit a distinctly different characteristics in terms of equilibrium distance and binding energy. The potentials reveal that O atoms come much closer to the Au(111) surface than C atoms and interact stronger. This behaviour is even enhanced in the parameter sets with lowest E_{ads} (thick, light coloured). The interaction in the adsorbed state (at the adsorption height), on the other hand, is generally very similar for both species. The difference between O_{an} and O_{carb} atoms as modeled by an exponential attractive potential applied only to the latter is small as indicated by the potential averaged over all best fits (purple).

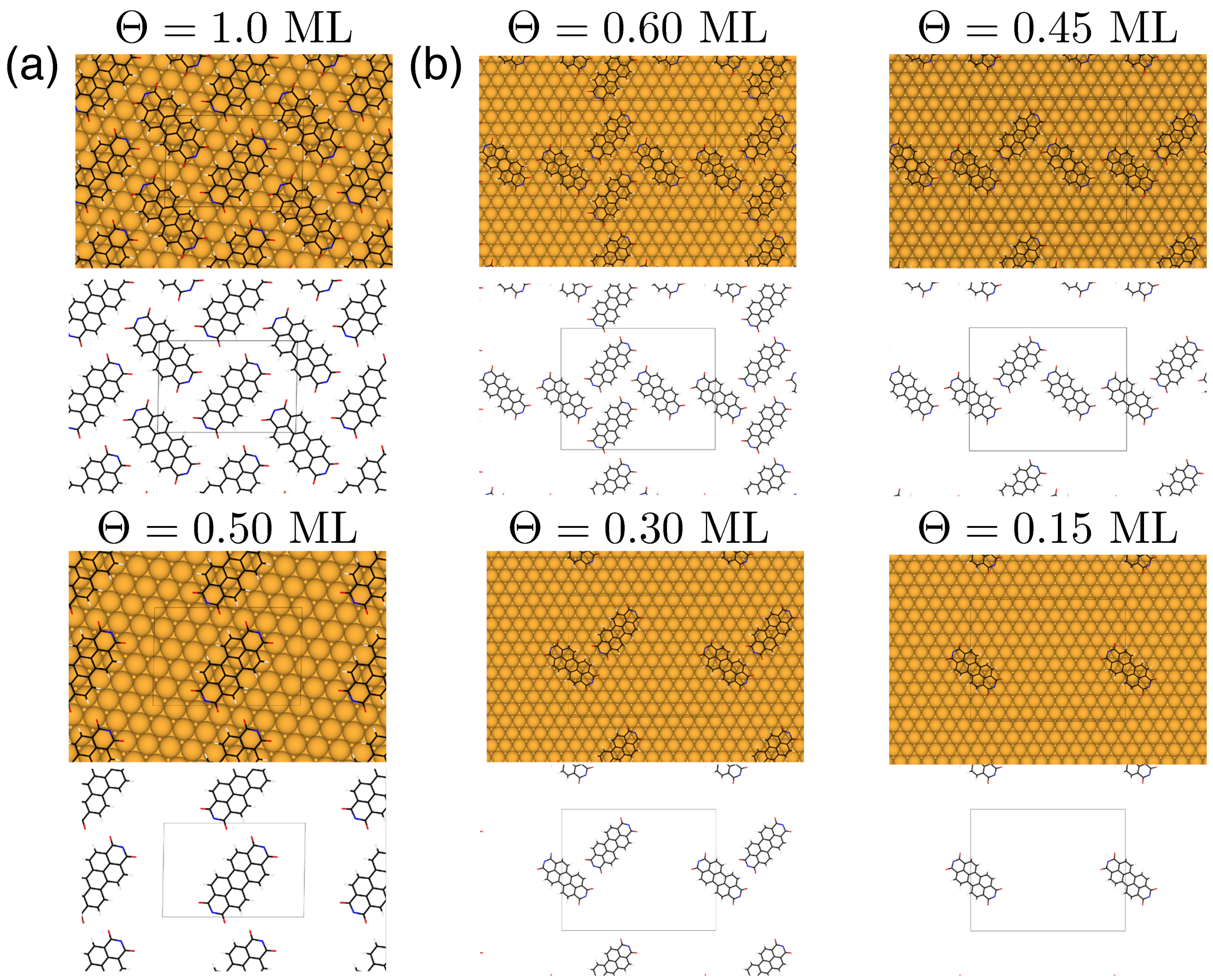

Fig. S2. Structural models for the adsorption of PTCDA on Au(111). (a) Structural models for molecule surface densities of 1.0 and 0.5 ML generated using a $\begin{pmatrix} 6 & 1 \\ -3 & 5 \end{pmatrix}$ surface unit cell consisting of five each with 33 Au atoms (40). (b) Structural models for molecule surface densities of 0.60, 0.45, 0.30, and 0.15 ML, generated using a slab with an orthorombic unit cell with vectors of magnitude $a = 32.35$ and $b = 25.47$ Å, and three layers for the substrate each with 110 atoms of Au. The substrates were built in both cases using the PBE lattice constant of Au, 4.159 Å (1). See Supplementary Methods for more details of the adsorption model.

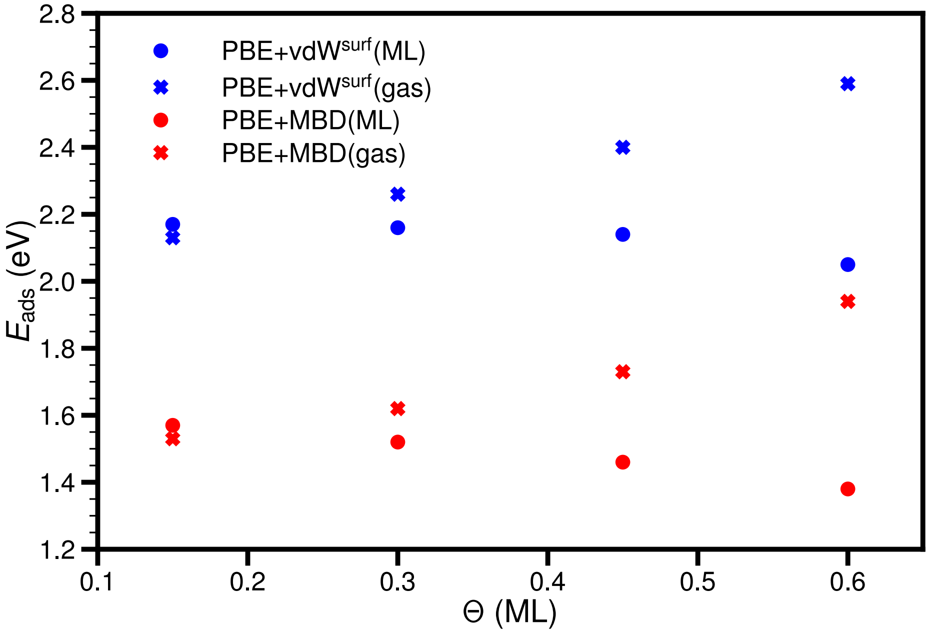

Fig. S3. Adsorption energy $E_{\mathrm{ads}}^{\Theta}$ using the gas phase molecule (gas) and the free-standing PTCDA surface density in PBC (ML) as references for PTCDA on Au(111) at molecule surface densities Θ of 0.60, 0.45, 0.30, and 0.15 ML with the PBE+vdW^{surf} and the PBE+MBD methods.

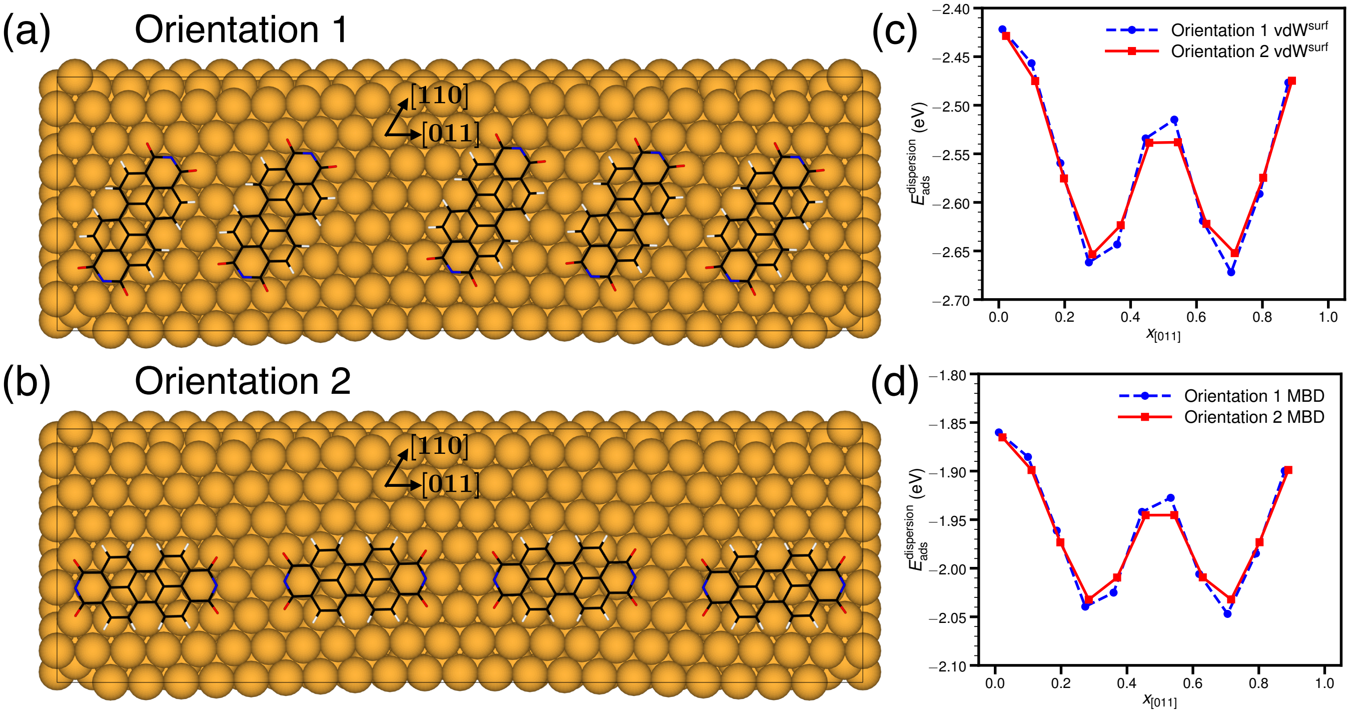

Fig. S4. Structural model for the adsorption energy of a single PTCDA molecule on the $(22 \times \sqrt{3})$ reconstruction of the Au(111) surface. A total of 22 molecular configurations were considered. (a) Structure 1 corresponds to the case in which the molecular axis is aligned paralell to the $[110]$ direction of the substrate. Five exemplary cases are shown from a total of eleven considered. (b) Structure 2 corresponds to the case in which the molecular axis is align parallel to the $[011]$ direction to the substrate. Four out of a total of eleven configurations are shown. (c) and (d) Dispersion contribution to the adsorption energy of a single PTCDA molecule on the reconstructed Au(111) surface. $x_{[011]}$ denotes the position of the center of the molecule in the [011] direction of the surface area in units of the lattice vector of the surface slab (i.e., spanning from 0.0 to 1.0). Energies for both structure 1 and 2 are shown using the vdW$^{\rm surf}$ (c) and MBD (d) methods.

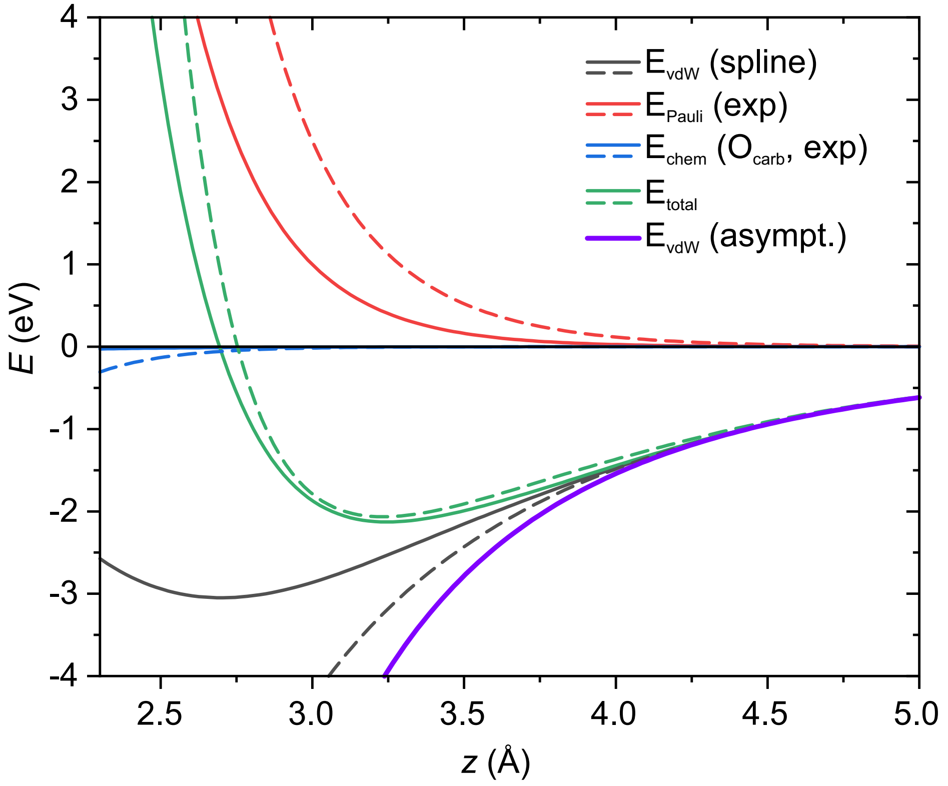

Fig. S5. Model of the molecule-surface potential. The total molecule-surface potential (green) is the sum of three contributions: The vdW potential (cubic spline, grey), the Pauli repulsion (exponential, red), and the chemical interaction of the O_{carb} atoms with the surface (exponential, blue). The three contributions cannot be interpreted individually, only the total potential is relevant. This becomes obvious when comparing the two given examples (solid lines, dashed lines) which both represent equally good fits of the experimental data (see Figure 2 of the main manuscript) achieved with strong (but largely canceling) differences in the vdW and Pauli repulsion potentials. For comparison, the purple curve shows the asymptotic vdW potential which is not valid for $z < 4.5$ Å (see text).

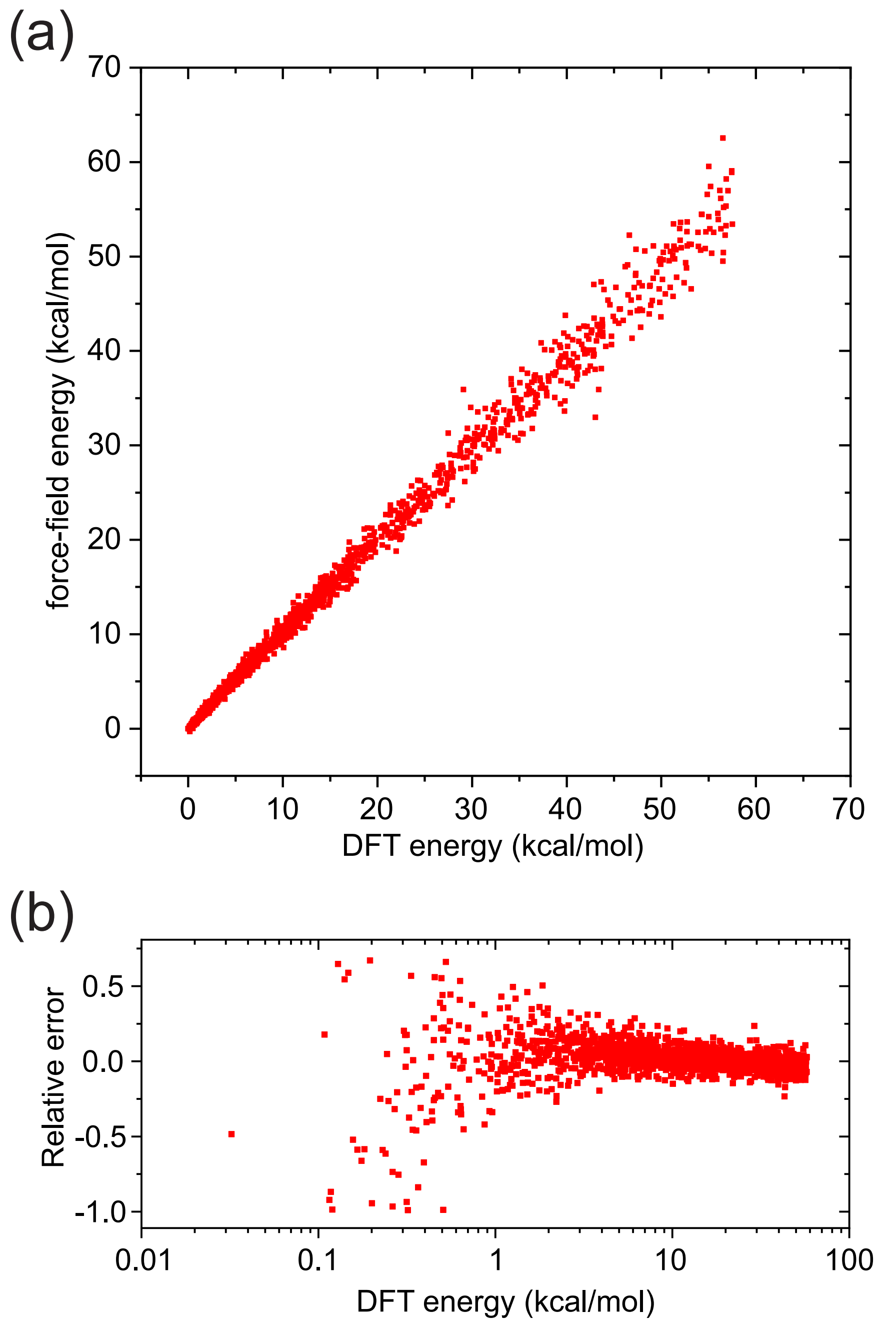

Fig. S6. Fit of intramolecular force field. (a) Plot of $E_{\mathrm{FF}} = \epsilon_{\mathrm{FF}} - \epsilon^0_{\mathrm{FF}}$ versus $E_{\mathrm{DFT}} = \epsilon_{\mathrm{DFT}} - \epsilon^0_{\mathrm{DFT}}$ for the optimised FF parameters for all 1500 conformations in the training set. ϵ^0_{FF} and $\epsilon^0_{\mathrm{DFT}}$ refer to the relaxed DFT gas-phase geometry of PTCDA. (b) Relative error $(E_{\mathrm{FF}} - E_{\mathrm{DFT}})/E_{\mathrm{DFT}}$ of the data in Panel a. A value below -1 means that the FF predicts the respective conformation to have lower energy than the relaxed DFT geometry (points not shown).

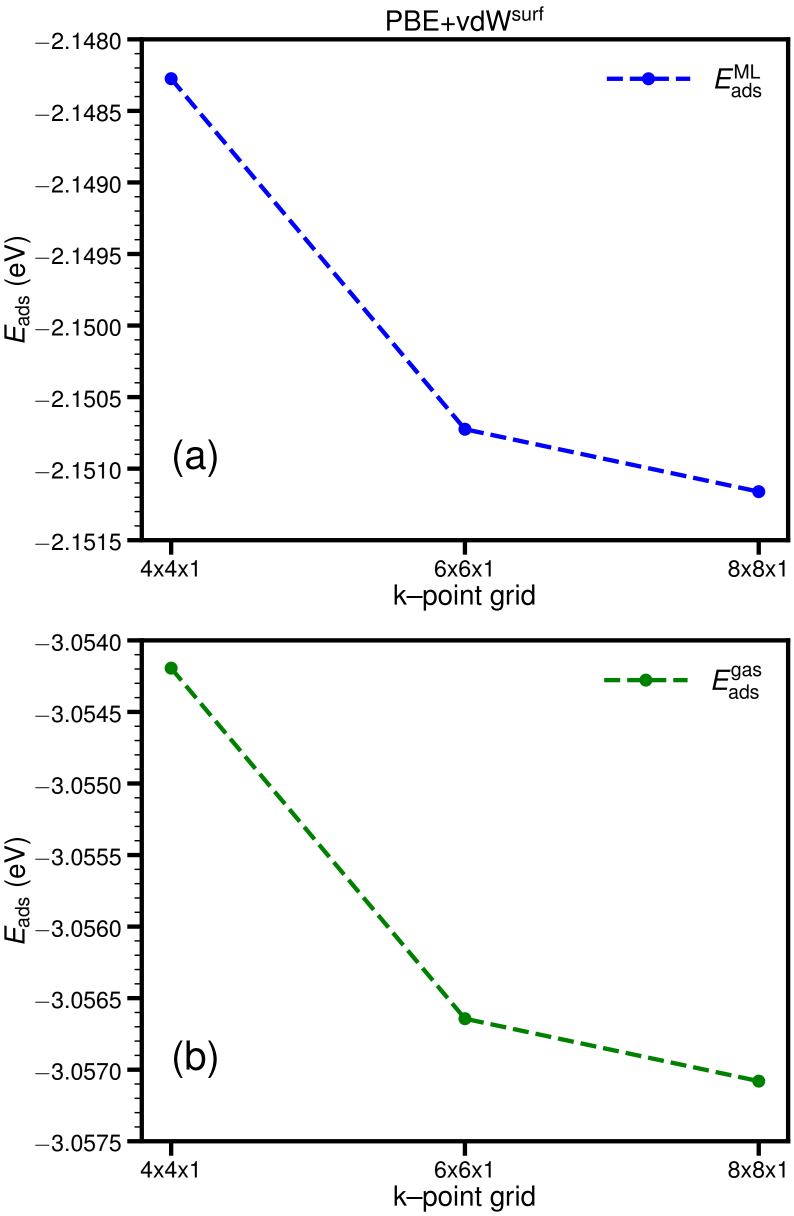

Fig. S7. Numerical convergence of the adsorption energy at full monolayer coverage ($\Theta = 1.0$ ML) with respect to the k–point grid of the DFT calculation using the PBE+vdW$^{\rm surf}$ method.

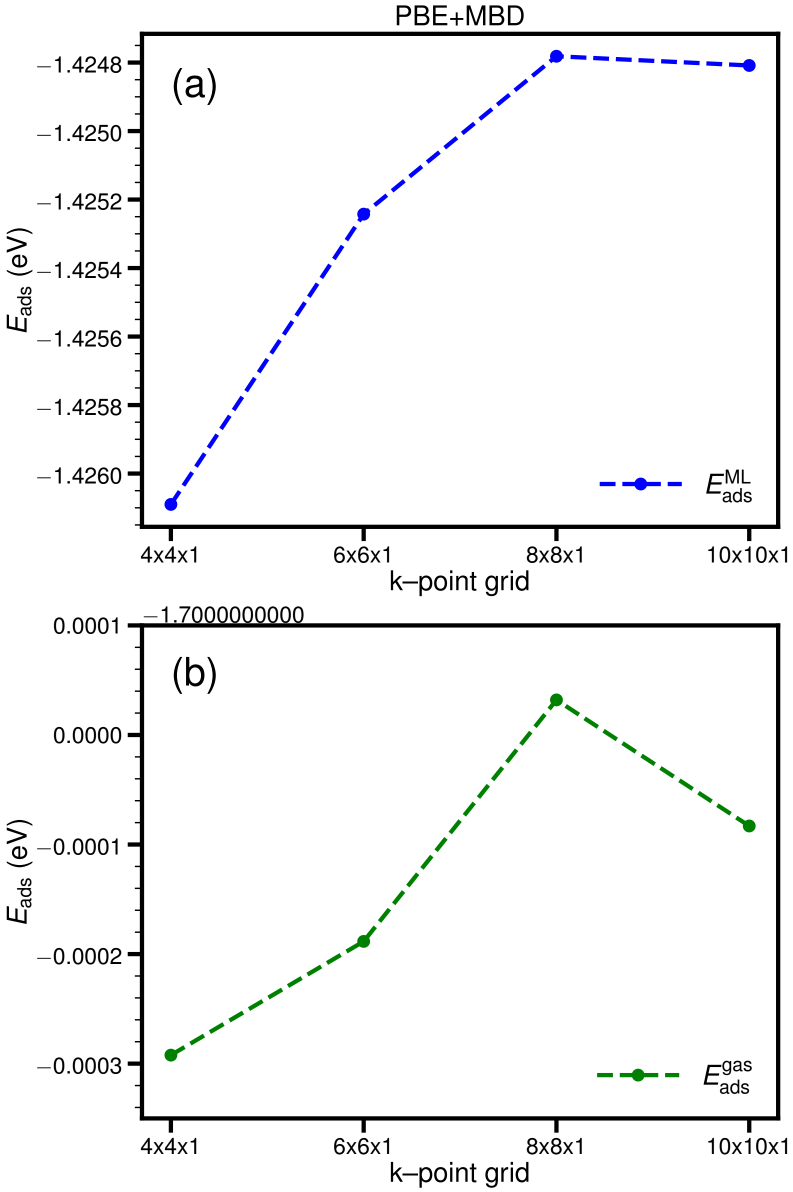

Fig. S8. Numerical convergence of the PBE+MBD adsorption energy at full monolayer coverage ($\Theta = 1.0$ ML) with respect to the k–point grid for the MBD energy calculation. The underlying PBE calculation corresponds to that one with a k–point grid of $6 \times 6 \times 1$.

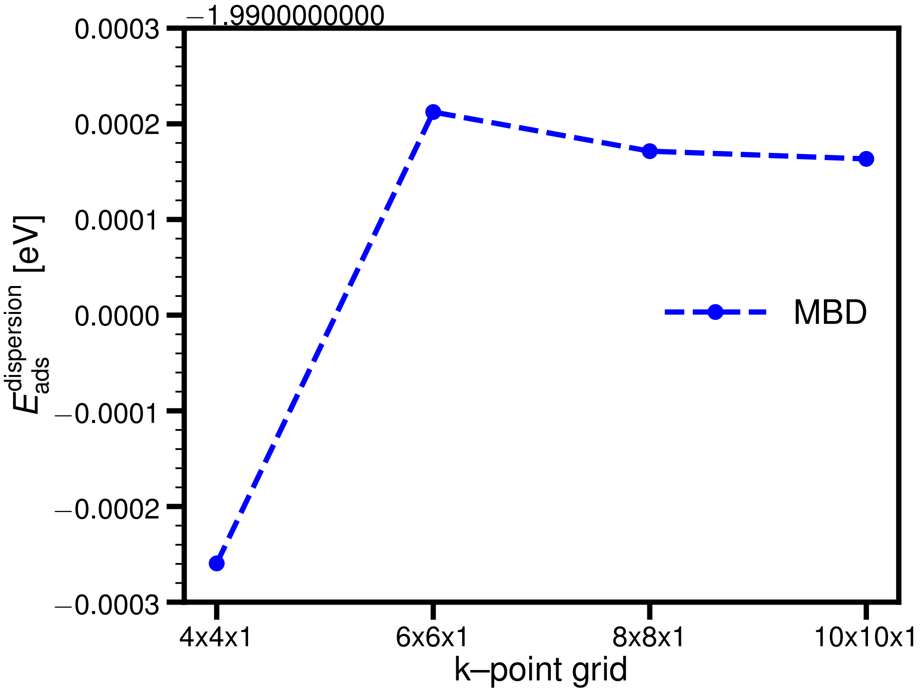

Fig. S9. Numerical convergence of the dispersion adsorption energies with respect to the k–point grid for the MBD calculation. The MBD energy calculations were performed using PBE Hirshfeld weights as input.

Table S1. Adsorption energy $\mathbf{E}_{\mathbf{ads}}^{\Theta}$ for PTCDA on Au(111) at a PTCDA surface density Θ of 1.0 and 0.5 ML using the PBE+vdW$^{\mathbf{surf}}$ and the PBE+MBD methods. An additional adsorption energy at $\Theta = 1.0$ ML using the PBE+MBD-NL functional is also shown as a comparison. The structure corresponds to the structural relaxation obtained with the PBE+vdW$^{\mathbf{surf}}$ method, modeled with a $\left(\begin{smallmatrix} 6 & 1 \\ -3 & 5 \end{smallmatrix}\right)$ surface unit cell consisting of five layers each with 33 Au atoms.

	$E_{\text{ads}}^{\Theta(\text{ML})}$ (eV)		$E_{\text{ads}}^{\Theta(\text{gas})}$ [eV]	
	1.0 ML	0.5 ML	1.0 ML	0.5 ML
PBE+vdW^{surf}	2.15	2.23	3.06	2.51
PBE+MBD	1.42	1.52	2.36	1.81
PBE+MBD-NL	1.43	–	2.33	–

Table S2. Adsorption energy $\mathbf{E}_{\mathbf{ads}}^{\boldsymbol{\Theta}}$ for PTCDA on Au(111) at PTCDA surface densities $\boldsymbol{\Theta}$ of 0.60, 0.45, 0.30, and 0.15 ML using the PBE+vdW$^{\mathbf{surf}}$ and the PBE+MBD methods. All calculations were performed using a $\mathbf{2 \times 2 \times 1}$ k–point grid except for one at $\boldsymbol{\Theta = 0.15}$ ML (see Supplementary Methods). The value at the single-molecule limit includes a correction of 0.08 and 0.05 eV for the PBE+vdW$^{\mathbf{surf}}$ and PBE+MBD methods, respectively, due to an underestimation originating in the number of subtrate layers for the metal surface.

Θ [ML]	PBE+vdW^{surf}		PBE+MBD	
	$E_{\mathrm{ads}}^{\Theta(\mathrm{ML})}$	$E_{\mathrm{ads}}^{\Theta(\mathrm{gas})}$	$E_{\mathrm{ads}}^{\Theta(\mathrm{ML})}$	$E_{\mathrm{ads}}^{\Theta(\mathrm{gas})}$
0.60	2.05	2.59	1.38	1.94
0.45	2.14	2.40	1.46	1.73
0.30	2.16	2.26	1.52	1.62
0.15	2.17	2.13	1.57	1.53
0.15 $(4 \times 4 \times 1)$	2.16	2.13	1.57	1.53
$\lim \Theta \to 0$ (corrected)	2.22		1.59	

Table S3. Adsorption energy $\mathbf{E^{\Theta}_{ads}}$ for PTCDA on Au(111) at a PTCDA surface density Θ of 1.0 ML using the PBE+vdW$^{\mathbf{surf}}$, PBE+MBD, HSE+vdW^{surf}, and the HSE+MBD methods. The structure corresponds to the structural relaxation obtained with the PBE+vdW$^{\mathbf{surf}}$ method, modeled with a $\left(\begin{smallmatrix} 6 & 1 \\ -3 & 5 \end{smallmatrix}\right)$ surface unit cell consisting of three metal layers. The value $\mathbf{\Delta_{HSE-PBE}}$ corresponds to the difference between the adsorption energy calculated with the HSE and PBE XC functionals. Details of the calculation settings can be found in the Supplementary Methods section.

(eV)	PBE+vdW^{surf}	HSE+vdW^{surf}	$\Delta_{\mathrm{HSE-PBE}}$	PBE+MBD	HSE+MBD	$\Delta_{\mathrm{HSE-PBE}}$
$E^{\Theta(\mathrm{ML})}_{\mathrm{ads}}$	2.20	2.44	0.24	1.49	1.74	0.25
$E^{\Theta(\mathrm{gas})}_{\mathrm{ads}}$	3.20	3.45	0.25	2.52	2.79	0.27

Table S4. Mean and standard deviation of the dispersion energy $\mathbf{E}_{\mathbf{ads}}^{\mathbf{dispersion}}$ (in eV) of 22 molecular configurations of a PTCDA molecule on Au(111) at a molecule surface density $\Theta \approx 0.09$ ML using the vdW^{surf} and MBD^{surf} methods. The substrates correspond to the (*a*) Au(111) surface modeled with a (14×13) unit cell and six metal layers and the (*b*) reconstructed $(22 \times \sqrt{3})$ Au(111) surface was modeled with a (1×4) surface unit cell consisting of six Au layers. $\mathrm{d}_{\mathrm{PTCDA}}$ denotes the distance (in Å) of the molecule with respect to the averaged position of the surface atomic top layer.

	(*a*) Au(111) Surface		(*b*) Reconstructed Au(111) Surface		$\Delta E_{\mathrm{ads}}^{\mathrm{dispersion}}$
	d_{PTCDA}	$E_{\mathrm{ads}}^{\mathrm{dispersion}}$	d_{PTCDA}	$E_{\mathrm{ads}}^{\mathrm{dispersion}}$	(*b*) − (*a*)
vdW^{surf}	3.19 ± 0.00	2.50 ± 0.00	3.19 ± 0.04	2.56 ± 0.08	[−0.02, 0.14]
MBD		1.92 ± 0.00		1.96 ± 0.06	[−0.02, 0.10]

Table S5. Dependence of the adsorption energy on the number atom layers for the surface slab for PTCDA on Au(111) at $\Theta = 1.0$ ML using the PBE+vdW^{surf} method. A relaxation of the interfaca has been performed for an interface with both three and five layers (see text). Using the relaxed structure of the interface with five layers, a single point calculation of an interface with six layers has also been perfomed. All energies are given in eV.

PBE+vdW^{surf}	Relaxation			Single point	
(eV)	3 layers	5 layers	5 layers − 3 layers	6 layers	6 layers − 5 layers
$E_{\mathrm{ads}}^{\Theta(\mathrm{ML})}$	2.07	2.15	0.08	2.18	0.03
$E_{\mathrm{ads}}^{\Theta(\mathrm{gas})}$	2.98	3.06	0.08	3.07	0.01

Table S6. Dependence of the adsorption energy on the number atom layers for the surface slab for PTCDA on Au(111) at $\Theta = 1.0$ ML using the PBE+MBD method. A relaxation of the interfaca has been performed for an interface with both three and five layers (see text). Using the relaxed structure of the interface with five layers, a single point calculation of an interface with six layers has also been perfomed. All energies are given in eV.

PBE+MBD	Relaxation			Single point	
(eV)	3 layers	5 layers	5 layers − 3 layers	6 layers	6 layers − 5 layers
$E_{\mathrm{ads}}^{\Theta(\mathrm{ML})}$	1.38	1.43	0.05	1.46	0.03
$E_{\mathrm{ads}}^{\Theta(\mathrm{gas})}$	2.31	2.36	0.05	2.37	0.01

Table S7. Adsorption energy $\mathbf{E}^{\Theta}_{\mathbf{ads}}$ for PTCDA on Au(111) at a coverage Θ of 0.15 ML using the PBE+vdW^{surf} and the PBE+MBD methods with respect to the k–point grid of the DFT calculation.

	$E^{\Theta(\mathrm{ML})}_{\mathrm{ads}}$ (eV)		$E^{\Theta(\mathrm{gas})}_{\mathrm{ads}}$ (eV)	
	$2 \times 2 \times 1$	$4 \times 4 \times 1$	$2 \times 2 \times 1$	$4 \times 4 \times 1$
PBE+vdW^{surf}	2.17	2.16	2.13	2.13
PBE+MBD	1.57	1.57	1.53	1.53

Table S8. Adsorption geometry of PTCDA on Au(111) at a surface density of 0.5 ML. We report the averaged vertical adsorption heights of the specific atoms obtained with PBE+vdW^{surf} calculations for three different Au(111) surfaces: a compression and an expansion of the surface unit cell labeled "Compression" and "Expansion", respectively. "Ideal" corresponds to a surface unit cell built using the PBE lattice constant. The adsorption height is given in Å with respect to the unrelaxed topmost metal layer for a direct comparison to NIXSW experiments. ΔA_{111} corresponds to the input surface unit cell of the adsorption system, while Δd_{12} corresponds to the resulting expansion or compression of the top two layers with respect to the ideal (111) surface. An estimated experimental adsorption height, which takes into account an estimated outward relaxation of the topmost metal layer by 3%, reduces the adsorption height of the carbon backbone to 3.27 Å.

	ΔA_{111}	Carbon backbone (Å)	Oxygen (Å)	Δd_{12}
Compression	−4.9%	3.52	3.52	5.2%
Ideal	0.0%	3.18	3.21	0.7%
Expansion	5.1%	2.84	2.82	−2.8%

Table S9. Adsorption energy of PTCDA on Au(111) at a surface density of 0.5 ML for three different Au(111) surfaces: a compression and an expansion of the surface unit cell labeled "Compression" and "Expansion", respectively. "Ideal" corresponds to a surface unit cell built using the PBE lattice constant. All energies are given in eV. The column "$\mathbf{E_{ads}}$ contributions" show the contributions from PBE and dispersion effects to the final adsorption energy. In our definition of the adsorption energy, negative values correspond to repulsive contributions while positive values are attractive. The column "$\mathbf{E_{ads}^{\Theta=0.5(ML)}}$" corresponds to final adsorption energies including both PBE and dispertion contributions.

	E_{ads} contributions			$E_{\text{ads}}^{\Theta=0.5(\text{ML})}$	
	PBE	vdW^{surf}	MBD	PBE+vdW^{surf}	PBE+MBD
Compression	-0.49	2.70	1.99	2.21	1.50
Ideal	-0.40	2.66	1.96	2.27	1.56
Expansion	-0.41	2.60	1.97	2.18	1.56

Supplementary References

1. W Liu, et al., Structure and energetics of benzene adsorbed on transition-metal surfaces: density-functional theory with van der Waals interactions including collective substrate response. *New J. Phys*. **15**, 053046 (2013).
2. H Ihm, HM Ajo, JM Gottfried, P Bera, CT Campbell, Calorimetric measurement of the heat of adsorption of benzene on pt(111). *The J. Phys. Chem. B* **108**, 14627–14633 (2004).
3. Q Ge, R Kose, DA King, Adsorption energetics and bonding from femtomole calorimetry and from first principles theory in *Impact of Surface Science on Catalysis*, Advances in Catalysis. (Academic Press) Vol. 45, pp. 207–259 (2000).
4. RJ Maurer, et al., Thermal and electronic fluctuations of flexible adsorbed molecules: azobenzene on ag(111). *Phys. Rev. Lett*. **116**, 146101 (2016).
5. M Miletic, K Palczynski, J Dzubiella, Quantifying entropic barriers in single-molecule surface diffusion. *The J. Chem. Phys*. **153**, 164713 (2020).
6. C Wagner, N Fournier, FS Tautz, R Temirov, Measurement of the binding energies of the organic-metal perylene-teracarboxylic-dianhydride/Au(111) bonds by molecular manipulation using an atomic force microscope. *Phys. Rev. Lett*. **109**, 076102 (2012).
7. C Wagner, et al., Non-additivity of molecule-surface van der Waals potentials from force measurements. *Nat. Commun*. **5**, 5568 (2014).
8. A Tkatchenko, M Scheffler, Accurate Molecular Van Der Waals Interactions from Ground-State Electron Density and Free-Atom Reference Data. *Phys. Rev. Lett*. **102**, 073005 (2009).
9. C Wagner, N Fournier, FS Tautz, R Temirov, The role of surface corrugation and tip oscillation in single-molecule manipulation with a non-contact atomic force microscope. *Beilstein J. Nanotechnol*. **5**, 202–209 (2014).
10. FJ Giessibl, A direct method to calculate tip–sample forces from frequency shifts in frequency-modulation atomic force microscopy. *Appl. Phys. Lett*. **78**, 123 (2001).
11. C Toher, et al., Electrical transport through a mechanically gated molecular wire. *Phys. Rev. B* **83**, 155402 (2011).
12. C Wagner, et al., Scanning Quantum Dot Microscopy. *Phys. Rev. Lett*. **115**, 026101 (2015).
13. RA DiStasio Jr., VV Gobre, A Tkatchenko, Many-body van der Waals interactions in molecules and condensed matter. *J. Physics: Condens. Matter* **26**, 213202 (2014).
14. VG Ruiz, W Liu, E Zojer, M Scheffler, A Tkatchenko, Density-Functional Theory with Screened van der Waals Interactions for the Modeling of Hybrid Inorganic-Organic Systems. *Phys. Rev. Lett*. **108**, 146103 (2012).
15. VG Ruiz, W Liu, A Tkatchenko, Density-functional theory with screened van der Waals interactions applied to atomic and molecular adsorbates on close-packed and non-close-packed surfaces. *Phys. Rev. B* **93**, 035118 (2016).
16. RJ Maurer, et al., Adsorption structures and energetics of molecules on metal surfaces: Bridging experiment and theory. *Prog. Surf. Sci*. **91**, 72 (2016).
17. VG Ruiz, Ph.D. thesis (Fritz-Haber-Institut der MPG) (2016).
18. A Tkatchenko, RA DiStasio Jr., R Car, M Scheffler, Accurate and Efficient Method for Many-Body van der Waals Interactions. *Phys. Rev. Lett*. **108**, 236402 (2012).
19. A Ambrosetti, AM Reilly, RA DiStasio Jr., A Tkatchenko, Long-range correlation energy calculated from coupled atomic response functions. *The J. Chem. Phys*. **140**, 18A508 (2014).
20. S Kümmel, L Kronik, Orbital-dependent density functionals: Theory and applications. *Rev. Mod. Phys*. **80**, 3 (2006).
21. J Heyd, GE Scuseria, M Ernzerhof. *J. Chem. Phys*. **118**, 8207 (2003).
22. J Heyd, GE Scuseria, M Ernzerhof. *J. Chem. Phys*. **124**, 219906(E) (2006).
23. MA van Hove, et al. *Surf. Sci*. **103**, 189 (1981).
24. F Hanke, J Björke. *Phys. Rev. B* **87**, 235422 (2013).
25. TT Schmitz-Hübsch, et al., Epitaxial growth of 3,4,9,10-perylene-tetracarboxylic-dianhydride on Au(111): A STM and RHEED study. *Phys. Rev. B* **55**, 7972 (1997).
26. S Mannsfeld, et al., Combined LEED and STM study of PTCDA growth on reconstructed Au(111) and Au(100)

single crystals. *Org. Electron.* **2**, 121 (2001).
27. L Kilian, E Umbach, M Sokolowski, A refined structural analysis of the PTCDA monolayer on the reconstructed Au(111) surface–"Rigid or distorted carpet?". *Surf. Sci.* **600**, 2633 (2006).
28. DA King, Thermal desorption from metal surfaces: A review. *Surf. Sci.* **47**, 384–402 (1975).
29. E Habenschaden, J Küppers, Evaluation of flash desorption spectra. *Surf. Sci.* **138**, L147–L150 (1984).
30. DLS Nieskens, AP Van Bavel, JW Niemantsverdriet, The analysis of temperature programmed desorption experiments of systems with lateral interactions; implications of the compensation effect. *Surf. Sci.* **546**, 159–169 (2003).
31. M Schulze, C Bronner, P Tegeder, Adsorption energetics of azobenzenes on noble metal surfaces. *J. Phys.: Condens. Matter* **26**, 355004 (2014).
32. C Wagner, R Temirov, FS Tautz, Perspectives of Molecular Manipulation and Fabrication in *Molecular Architectonics The Third Stage of Single Molecule Electronics*, ed. T Ogawa. (Springer Nature, Cham, Switzerland), (2017).
33. S Henze, O Bauer, TL Lee, M Sokolowski, F Tautz, Vertical bonding distances of PTCDA on Au(111) and Ag(111): Relation to the bonding type. *Surf. Sci.* **601**, 1566–1573 (2007).
34. EM Lifshitz, The Theory of Molecular Attractive Forces between Solids. *Sov. Physics-Journal Exp. Theor. Phys.* **2**, 73 (1956) original version (russian): ZhETF **29**, 94, (1956).
35. E Zaremba, W Kohn, Van der Waals interaction between an atom and a solid surface. *Phys. Rev. B* **13**, 2270 (1976).
36. A Tkatchenko, A Ambrosetti, R DiStasio Jr., Interatomic methods for the dispersion energy derived from the adiabatic connection fluctuation-dissipation theorem. *The J. Chem. Phys.* **138**, 074106 (2013).
37. A Ambrosetti, D Alfe, RA DiStasio Jr., A Tkatchenko, Hard Numbers for Large Molecules: Toward Exact Energetics for Supramolecular Systems. *The J. Phys. Chem. Lett.* **5**, 849 (2014).
38. J Hermann, A Tkatchenko, Density functional model for van der waals interactions: Unifying many-body atomic approaches with nonlocal functionals. *Phys. Rev. Lett.* **124**, 146401 (2020).
39. P Fenter, F Schreiber, L Zhou, P Eisenberger, S Forrest, In situ studies of morphology, strain, and growth modes of a molecular organic thin film. *Phys. Rev. B* **56**, 3046 (1997).
40. L Romaner, D Nabok, P Puschnig, E Zojer, C Ambrosch-Draxl, Theoretical study of PTCDA adsorbed on the coinage metal surfaces, Ag(111), Au(111) and Cu(111). *New J. Phys.* **11**, 053010 (2009).
41. HJ Monkhorst, JD Pack, Special points for Brillouin-zone integrations. *Phys. Rev. B* **13**, 5188 (1976).
42. KG Huang, D Gibbs, DM Zehner, AR Sandy, SGJ Mochrie. *Phys. Rev. Lett.* **65**, 3313 (1990).
43. AR Sandy, SGJ Mochrie, DM Zehner, KG Huang, D Gibbs. *Phys. Rev. B* **43**, 4667 (1991).
